# All-optical image classification through unknown random diffusers using a single-pixel diffractive network

**DOI:** 10.1038/s41377-023-01116-3

**Published:** 2023-03-09

**Authors:** Bijie Bai, Yuhang Li, Yi Luo, Xurong Li, Ege Çetintaş, Mona Jarrahi, Aydogan Ozcan

**Affiliations:** 1 Electrical and Computer Engineering Department, University of California, Los Angeles, California, 90095 USA; 2 Bioengineering Department, University of California, Los Angeles, California, 90095 USA; 3 California Nano Systems Institute (CNSI), University of California, Los Angeles, California, 90095 USA

**Keywords:** Imaging and sensing, Optical physics

## Abstract

Classification of an object behind a random and unknown scattering medium sets a challenging task for computational imaging and machine vision fields. Recent deep learning-based approaches demonstrated the classification of objects using diffuser-distorted patterns collected by an image sensor. These methods demand relatively large-scale computing using deep neural networks running on digital computers. Here, we present an all-optical processor to directly classify unknown objects through unknown, random phase diffusers using broadband illumination detected with a single pixel. A set of transmissive diffractive layers, optimized using deep learning, forms a physical network that all-optically maps the spatial information of an input object behind a random diffuser into the power spectrum of the output light detected through a single pixel at the output plane of the diffractive network. We numerically demonstrated the accuracy of this framework using broadband radiation to classify unknown handwritten digits through random new diffusers, never used during the training phase, and achieved a blind testing accuracy of 87.74 ± 1.12%. We also experimentally validated our single-pixel broadband diffractive network by classifying handwritten digits “0” and “1” through a random diffuser using terahertz waves and a 3D-printed diffractive network. This single-pixel all-optical object classification system through random diffusers is based on passive diffractive layers that process broadband input light and can operate at any part of the electromagnetic spectrum by simply scaling the diffractive features proportional to the wavelength range of interest. These results have various potential applications in, e.g., biomedical imaging, security, robotics, and autonomous driving.

## Introduction

Imaging and recognizing objects through scattering media have been challenging in many fields, including biomedical imaging^1,2^, oceanography^3,4^, security^5^, robotics^6^, and autonomous driving^7,8^, among others^9^. Numerous computational solutions have been developed to reconstruct an image distorted by a diffuser: deconvolution algorithms were used when the transmission matrix of a diffuser can be premeasured as prior information^10^; adaptive optics and wavefront shaping methods were used with the help of guide-stars or reference objects^11,12^; iterative algorithms were used to solve for the images of the hidden objects utilizing the memory effect of a diffuser^13,14^. Multispectral or time-gated imaging methods were also used to bring additional degrees of freedom to recover the hidden objects^15,16^, and similarly, deep neural networks were used to learn the features of diffusers and generalize to see through them^17–22^. Despite their success, for each object to be imaged, all these methods require access to large-scale computing provided by digital computers for each blind inference event, which also hinders the practical frame rate of these computational imaging modalities. Furthermore, additional energy is consumed on the downstream tasks such as object recognition and image classification. Partially motivated by the fact that merging the two steps (image reconstruction and classification) could potentially reduce energy consumption and computing time, deep learning-based digital solutions to directly classify objects hidden behind scattering media have also been demonstrated^23–25^, which predicted the object class using speckle patterns as inputs without any digital image reconstruction. Although deep learning-based methods have the generalization capability to image through unseen diffusers^19–22^, for the direct classification of input objects distorted by random diffusers, existing methods lack generalization to blind, new diffusers that were never used in the training phase.

Recent works have presented an all-optical method to image through unknown diffusers using diffractive deep neural networks (D^2^NNs), enabling passive, computer-free image reconstruction at the speed of light propagation through thin optical layers^26,27^. Diffractive networks form an all-optical machine learning platform that computes a given task using light diffraction through successive transmissive layers^28^. Each diffractive layer typically consists of tens of thousands of diffractive units (termed “diffractive neurons”) that modulate the phase and/or amplitude of the incident light. Deep learning tools, such as error back-propagation, are used to optimize the modulation values (e.g., transmission coefficients) of each layer, mapping a complex-valued input field containing the optical information of interest (to-be-processed) onto a desired output field. Computing using diffractive networks possesses the benefits of high speed, parallelism, and low power consumption: the computational task of interest is completed while the incident light passes through passive thin diffractive layers at the speed of light, requiring no energy other than illumination. This framework’s success and capabilities were demonstrated numerically and experimentally by achieving various computational tasks, including object classification^28–31^, hologram reconstruction^32^, quantitative phase imaging^33^, privacy-preserving class-specific imaging^34^, logic operations^35,36^, universal linear transformations^37^, and polarization processing^38^, among others^39–48^. Diffractive networks can also process and shape the phase and amplitude of broadband input spectra to perform various tasks such as pulse shaping^49^, wavelength-division multiplexing^50^, and single-pixel image classification^51^.

**Fig. 1:**
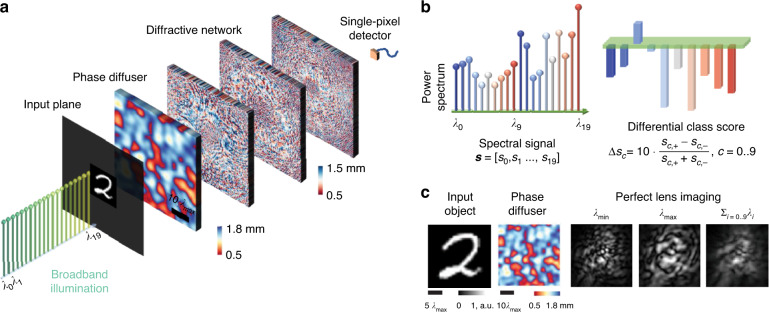
A single-pixel broadband diffractive neural network classifies handwritten digits through unknown random diffusers. **a** The schematic drawing of a broadband single-pixel diffractive network mapping the spatial information of an input handwritten digit behind an unknown diffuser into the power spectrum at the output pixel aperture. **b** Differential spectral class score encoding scheme using 20 illumination wavelengths. **c** Visualization of the level of image distortions through a random phase diffuser with a correlation length of ~25 $\lambda _{\max }$. The distortions induced by the random diffuser are severe at all the utilized wavelengths between $\lambda _{\min }$ and $\lambda _{\max }$

Here, we demonstrate broadband diffractive networks to directly classify unknown objects (e.g., MNIST handwritten digits^52^) through unknown, random diffusers using a single-pixel spectral detector (Fig. 1a). This broadband diffractive architecture uses 20 discrete wavelengths, mapping a diffuser-distorted complex optical field containing the spatial information of an input object into a spectral signature detected through a single pixel. A differential detection scheme was applied to the single-pixel output spectrum by assigning the intensities of 10 predetermined wavelengths as the positive scores and the intensities of the remaining 10 predetermined wavelengths as the negative scores, revealing the *differential spectral class scores* used for image classification through a single-pixel (Fig. 1b). During each training epoch, ***n*** different random phase diffusers with the same correlation length were used to generate unique distortions to the input objects. A loss function that penalized the classification accuracy through these random diffusers was used to optimize the modulation values on each diffractive layer. After being trained for 100 epochs, the single-pixel diffractive network successfully generalized to directly classify unknown handwritten digits completely hidden by unknown random phase diffusers never seen before during the training. After this one-time training, the resulting diffractive layers can be physically fabricated to form a passive single-pixel network that computes the desired classification task using only the illumination light without a digital computer. In our numerical simulations, this single-pixel broadband diffractive network achieved a blind testing accuracy of 87.74 ± 1.12%, successfully classifying handwritten digits through 80 randomly selected unknown phase diffusers, each with a correlation length of ~25 $\lambda _{\max }$, where $\lambda _{\max }$ is the longest wavelength used in the illumination spectrum. Furthermore, we experimentally demonstrated the capability of all-optical image classification through a random diffuser using a terahertz time-domain spectroscopy (THz-TDS) system and a 3D-printed diffractive network. To the best of our knowledge, the presented work constitutes the first demonstration of all-optical classification of objects through random diffusers that generalizes to unseen/new diffusers. The presented single-pixel diffractive designs that generalized to classify objects through unknown random diffusers can operate at any part of the electromagnetic spectrum (without the need for redesigning or retraining) by simply scaling the size of the diffractive features with respect to the operational wavelength range of interest.

Single-pixel all-optical diffractive image classification through random new diffusers presents a time- and energy-efficient solution for sensing and image classification through scattering media, with numerous potential applications in different fields, such as surveillance cameras, biomedical imaging, and autonomous driving.

## Results

### Design of a broadband single-pixel diffractive network to classify handwritten digits through random unknown diffusers

**Fig. 2:**
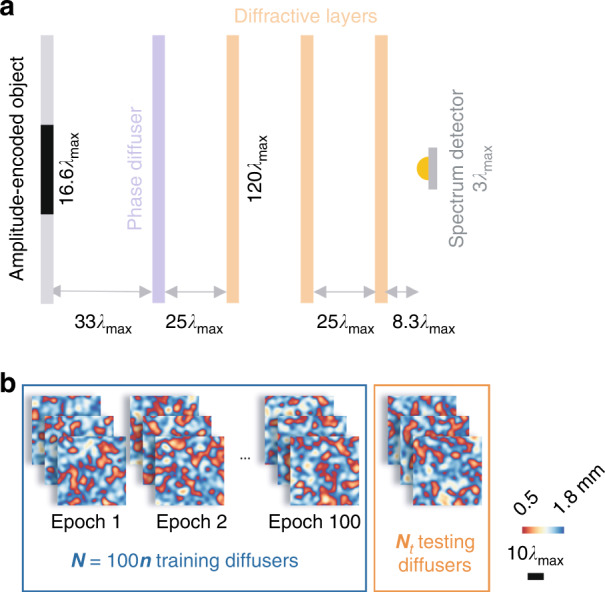
Training of a single-pixel broadband diffractive network. **a** The physical design of the diffractive network. **b** During each training epoch, ***n*** different random diffusers with the same correlation length were used to generate unique distortions to the input objects; ***N*** = 100***n*** training random diffusers are used across 100 epochs. *N*_*t*_ = 80 new random diffusers (never used in training) were used to verify the network’s generalization performance after the training

Broadband single-pixel diffractive networks were designed to classify MNIST handwritten digits placed behind unknown random diffusers using three successive diffractive layers and 20 discrete illumination wavelengths uniformly selected between $\lambda _{\min } = 0.6\,{{{\mathrm{mm}}}}$ and $\lambda _{\max } = 1.2\,{{{\mathrm{mm}}}}$. The spatial information of each handwritten digit placed at the input plane was encoded into the amplitude channel of all 20 wavelengths. A random phase diffuser with a correlation length of ~25 $\lambda _{\max }$ was placed 33 $\lambda _{\max }$ away from the input object along the optical axis to create random distortions to the optical field (Fig. 2a). The distances from the random diffuser to the first diffractive layer and between two successive diffractive layers were set to be ~25 $\lambda _{\max }$. The level of image distortion due to a random diffuser (with a correlation length of ~25 $\lambda _{\max }$) is visualized in Fig. 1c for $\lambda _{\min }$ and $\lambda _{\max }$, separately, as well as for all the 20 illumination wavelengths simultaneously on, shown for comparison. More examples of input images distorted by ~25 $\lambda _{\max }$ diffusers can be found in Supplementary Fig. S1.

Each distorted optical field at a given wavelength was forward propagated through three successive diffractive layers, each composed of 200 × 200 diffractive neurons that modulated the phase of the optical field at their corresponding locations. The transmission modulation of each neuron was determined by the dispersion of the diffractive material and its physical height (see the “Methods” section), which was optimized using deep learning and error back-propagation^28,30^.

A single square pixel with a width of 3 $\lambda _{\max }$ was placed 8.3 $\lambda _{\max }$ away from the last diffractive layer, measuring the intensity of all the 20 predetermined wavelengths. This can be achieved sequentially by, e.g., wavelength scanning or turning different sources on/off; alternatively, it can run simultaneously by having a spectroscopic detector behind the single-pixel aperture. The measured single-pixel power spectrum ${{{\boldsymbol{s}}}} = \left[ {s_0,s_1, \ldots s_{19}} \right]$ for these 20 wavelengths was paired in groups of two to differentially represent each spectral class score^29,51^. The first 10 spectral measurements at the single-pixel detector ($s_0$, $s_1$, …, $s_9$) were virtually assigned to be the positive signals ($s_{0, + }$, $s_{1, + }$, …, $s_{9, + }$), and the subsequent 10 spectral measurements ($s_{10}$, $s_{11}$, …, $s_{19}$) were virtually assigned to be the negative signals ($s_{0, - }$, $s_{1, - }$, …, $s_{9, - }$). Based on this, the differential spectral class score ${\Delta}s_{{{\boldsymbol{c}}}}$ for a given data class *c* can be defined as:

1 $$\begin{array}{*{20}{c}} {{\Delta}s_c = \frac{1}{T} \cdot \frac{{s_{c, + } - s_{c, - }}}{{s_{c, + } + s_{c, - }}}} \end{array}$$

where *T* is a fixed parameter set as 0.1. A *max(.)* operation on ${\Delta}s_c$ infers the final classification decision for the input object (see the “Methods” section for details).

The deep learning-based training process enables the diffractive networks to classify input objects through random unknown diffusers. Each training iteration starts with randomly selecting ***B*** = 4 digits from the MNIST training dataset (containing 50,000 handwritten digits) to form a training batch. The optical fields of the objects in each batch were independently propagated (at the 20 wavelengths of interest) to a phase-only random diffuser. The distorted fields were further propagated, modulated by three successive diffractive layers, and reached the single-pixel detector at the output. A softmax cross-entropy loss^30^ was calculated using the differential spectral class scores (${\Delta}s_c$) and the ground truth class labels to update the neurons’ height profiles through error back-propagation, which concluded one training batch. A training epoch finished when all the 50,000 training handwritten digits were used, i.e., after 12,500 batches. Within each epoch, ***n*** different random phase diffusers were used to ensure generalization to classify new test objects through unseen, new diffusers (Fig. 2b). Therefore, the random diffuser in the forward model was regularly updated after every ~12,500/***n*** training batches within each epoch. Each diffractive network was trained for 100 epochs; during its training, each diffractive network “saw” ***N*** = 100***n*** different random diffusers (referred to as *known* diffusers).

**Fig. 3:**
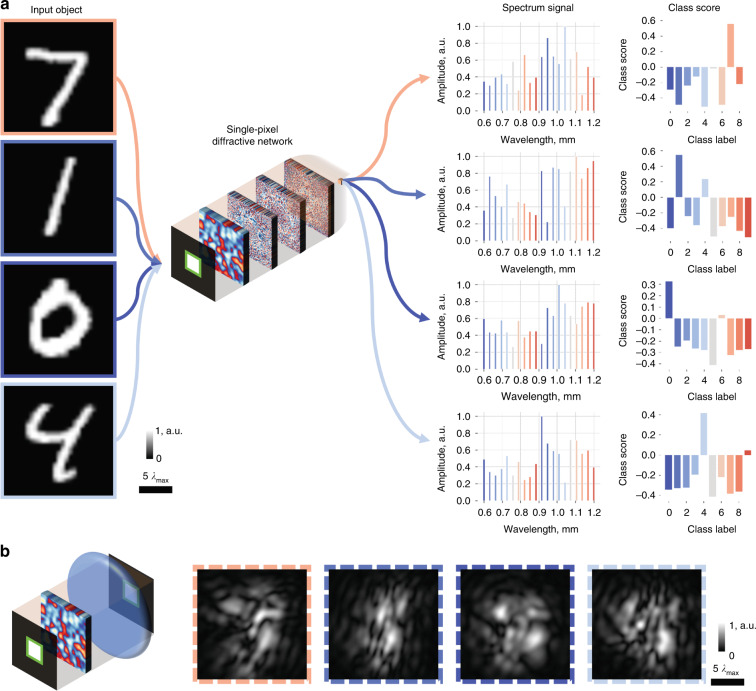
Examples of blind testing results of the presented single-pixel broadband diffractive network. **a** Example results for classifying handwritten digits through a new random diffuser using the single-pixel broadband diffractive network. **b** Comparison images for each test object of (**a**), seen through the same random diffuser using a diffraction-limited lens at $\lambda _{\max }$ illumination (0.8 NA). The spatial information of the input objects is severely distorted, making them unrecognizable

The trained single-pixel diffractive network is able to blindly classify handwritten objects through not only the diffusers used during the training (i.e., the known diffusers) but also new, random phase diffusers that were never seen by the network (see Fig. 3). To demonstrate this, we repeated the same training and blind testing process five times to report the average classification accuracy and standard deviation values for each model. For example, the diffractive network trained with ***n*** = 80 and ***N*** = 8000 different random diffusers achieved an average blind testing accuracy of 91.98 ± 0.24% classifying handwritten test digits through the same 80 known diffusers used in the *last training epoch;* the same single-pixel broadband diffractive network achieved an average blind testing accuracy of 87.74 ± 1.12% classifying handwritten test digits through *N*_*t*_ = 80 new random phase diffusers, never used in the training phase. This reduction in the handwritten digit classification accuracy of the diffractive network through new random diffusers, compared to the known diffusers used in the last epoch, indicates that the network overfitted to, or “memorized,” the random diffusers used in the last epoch. To shed more light on this, we further divided the known random diffusers into two categories: the *memorized* diffusers are the ***n*** random diffusers used in the last epoch of the training (epoch 100), and the *forgotten* random diffusers are those used in the training epochs 1–99 (except the last epoch). In terms of the input object classification performance, the single-pixel diffractive network treats the earlier training diffusers the same as the new ones: the single-pixel broadband diffractive network achieved a blind testing classification accuracy of $Acc_m$ = 91.98 ± 0.24% through 80 memorized random diffusers used in epoch 100, $Acc_f$ = 88.79 ± 0.23% through 7920 forgotten diffusers used in epochs 1–99, and $Acc_{new}$ = 87.74 ± 1.12% through 80 new random diffusers never used in the training phase. In addition, the classification accuracy of the same diffractive network when the random diffusers were removed was calculated to be $Acc_0$ = 96.29 ± 0.35%.

**Fig. 4:**
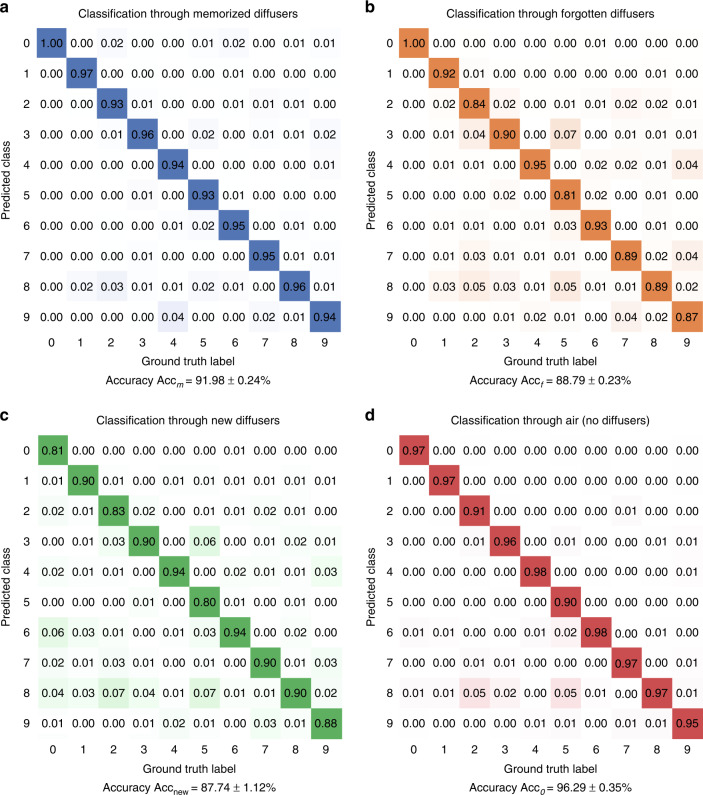
Classification results of the single-pixel broadband diffractive network through different types of random diffusers. Confusion matrices of the single-pixel broadband diffractive network trained with ***n*** = 80 (***N*** = 8000) random diffusers classifying handwritten test digits through **a** 80 memorized random diffusers used in the last training epoch, **b** 7920 forgotten random diffusers used in training epochs 1–99, and **c** 80 new random diffusers that were never used in the training of the diffractive network. **d** The confusion matrix of the same single-pixel broadband diffractive network, classifying distortion-free objects with no diffusers present. Notice that $Acc_0 > Acc_m > Acc_f$ and $Acc_f \approx Acc_{new}$. Since $Acc_f \approx Acc_{new}$, we conclude that the diffractive network “forgets” the random training diffusers used in epochs 1–99 and statistically treats them the same as a fresh random diffuser never used during the training

From these analyses, we conclude that $Acc_0 > Acc_m > Acc_f \approx Acc_{new}$, which indicates that (1) the single-pixel broadband diffractive network trained with random phase diffusers can classify input objects more accurately when there are no diffusers in the testing phase, showing that it converged to a decent single-pixel image classifier; (2) it partially memorized the random diffusers of the last epoch and performed better all-optical image classification through these memorized diffusers compared to the forgotten diffusers of the previous epochs; and (3) it performed at a similar level of classification accuracy for new random phase diffusers when compared to the forgotten diffusers since $Acc_f \approx Acc_{new}$. This brings more meaning to the term “forgotten diffuser” as it is statistically equivalent to a new random diffuser from the perspective of the broadband diffractive network’s image classification performance. Figure 4 supports the same conclusions, reporting the confusion matrices for diffractive single-pixel image classification without a diffuser as well as through the memorized, forgotten, and new random unknown diffusers.

**Fig. 5:**
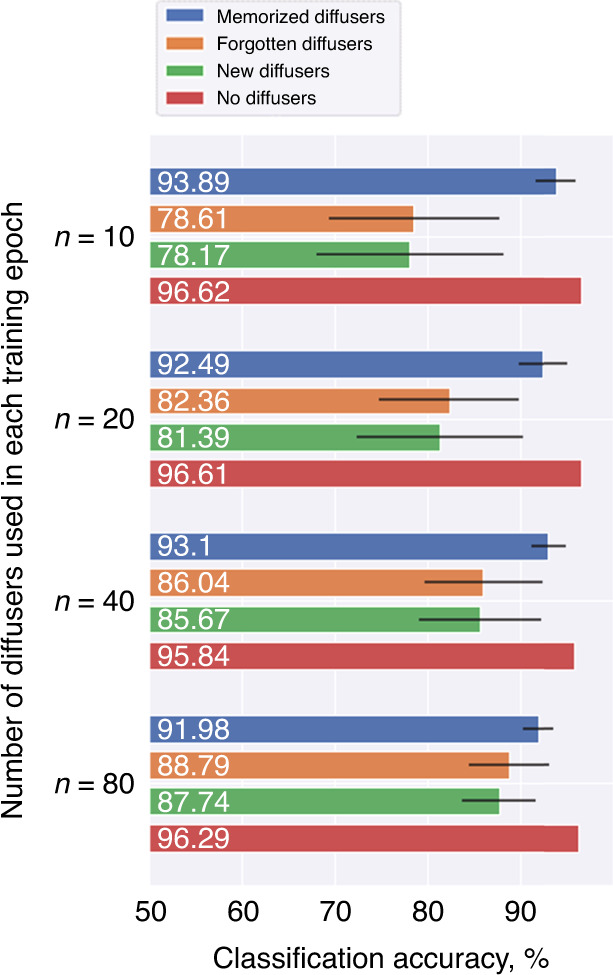
Impact of the number of independent diffusers used in each training epoch on the classification accuracy. Training with an additional number of random diffusers in each epoch improved the network’s generalization for classifying handwritten digits behind unknown random diffusers using a single output pixel

The single-pixel broadband diffractive network’s generalization capability, or its resilience to random new diffusers’ distortions, strongly correlates with the number of diffusers used in its training. To better highlight this feature, we further trained three additional single-pixel diffractive networks with ***n*** = 10, 20, and 40 random diffusers in each epoch (i.e., ***N*** = 1000, 2000, and 4000, respectively), and the resulting $Acc_m$, $Acc_f$, $Acc_{new}$, and $Acc_0$ values are compared in Fig. 5. With ***n*** = 10 (***N*** = 1000), the diffractive network obtained a strong memory for classification through diffusers, yielding $Acc_m$ = 93.89 ± 2.19%. However, the generalization capability was consequently limited, with $Acc_{new}$ = 78.17 ± 10.13%. An improved generalization over unknown random diffusers can be obtained when the network is trained with an increased number of diffusers in each epoch. For example, $Acc_{new}$ values increased to 81.39 ± 9.03%, 85.67 ± 6.58%, and 87.74 ± 1.12% when ***n*** = 20, 40, and 80, respectively. At the same time, the capability to classify objects without diffusers remained largely unchanged, with $Acc_0$ being 96.62% for ***n*** = 10 and 96.29% for ***n*** = 80. These results indicate that the diffractive network trained with a larger ***n*** learned the image classification task through random new diffusers better, converging to a state where more of the diffractive features were utilized to accommodate for the existence of a random phase diffuser for correct image classification through a single-pixel output detector.

### Image classification through random unknown diffusers with different correlation lengths

**Fig. 6:**
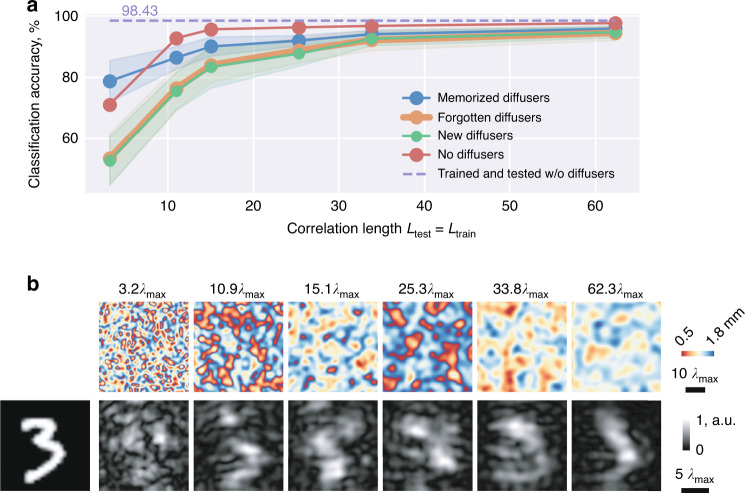
Single-pixel broadband diffractive networks trained to classify handwritten digits through random diffusers with different correlation lengths ($L_{train}$). **a** The classification accuracy of single-pixel broadband diffractive networks trained with different $L_{train}$, classifying handwritten test digits through memorized, forgotten, new, and no diffusers. The dashed purple line indicates the classification accuracy without any diffusers being present using a broadband diffractive network trained *without* any diffusers. **b** Comparison images of a test object seen through random diffusers with different correlation lengths (from 3.2 $\lambda _{\max }$ to 62.3 $\lambda _{\max }$) using a diffraction-limited lens at $\lambda _{\max }$ illumination. The spatial information of the input object is severely distorted in each case, making the object unrecognizable

To demonstrate the applicability of the presented framework under different levels of image distortion, we further trained five new diffractive networks with different correlation lengths, i.e., we used an $L_{train}$ of 3.2 $\lambda _{\max }$, 10.9 $\lambda _{\max }$, 15.1 $\lambda _{\max }$, 33.8 $\lambda _{\max }$, and 62.3 $\lambda _{\max }$. All these single-pixel broadband diffractive networks were trained following the same workflow as depicted in Fig. 2, only changing the random phase diffusers to create different levels of image distortions. Each one of the trained diffractive networks was separately tested with $N_t$ = 80 random unknown diffusers with $L_{test}$ = $L_{train}$ (see Fig. 6). Our results indicate that the single-pixel image classification networks that were trained and tested with random phase diffusers with larger correlation lengths achieved better classification accuracies, as shown in Fig. 6a. This improvement is largely owing to the reduced distortion generated by diffusers with a larger correlation length (see Fig. 6b). For each one of the diffractive networks shown in Fig. 6a, the image classification accuracies through random diffusers once again confirmed that $Acc_m > Acc_f$ and $Acc_f \approx Acc_{new}$, which were all lower than $Acc_0$. In fact, $Acc_0$ was also lower than the classification accuracy of a single-pixel broadband diffractive network that was trained and tested *without* any diffusers, which scored a blind testing accuracy of 98.43% in classifying distortion-free handwritten digits (see the dashed line in Fig. 6a).

It is also worth noting that $Acc_0$ experienced a relatively steep increase with larger correlation lengths. For example, the single-pixel broadband diffractive network designed for classifying input objects through $L_{train}$ = 3.2 $\lambda _{\max }$ random diffusers achieved a blind testing accuracy of $Acc_0$ = 71.11%, which drastically improved to 92.71% for $L_{train}$ = 10.9 $\lambda _{\max }$ and further increased to 97.67% for $L_{train}$ = 62.$3\lambda _{\max }$. This performance increase indicates that the single-pixel broadband diffractive networks trained with random phase diffusers present a trade-off between their image distortion resilience and diffuser-free image classification. To classify objects through random diffusers with smaller correlation lengths, the diffractive networks, in general, use more of their information processing capacity and degrees of freedom to mitigate the stronger distortions introduced by these random diffusers with finer grain size, intuitively resulting in a limited model capacity for the all-optical image classification task, which points to a trade-off between the random diffuser correlation length and the accuracy of the all-optical image classification task. With an increased correlation length, however, random diffusers create less distortions to the input optical fields, giving the diffractive networks more degrees of freedom to optimize their diffractive neurons for enhancing their diffuser-free image classification performance, i.e., $Acc_0$. That is why, $Acc_0$ increased to 97.67% and 92.71% from 71.11% when $L_{train}$ increased to 62.3 $\lambda _{\max }$ and 10.9 $\lambda _{\max }$ from 3.2 $\lambda _{\max }$, respectively (Fig. 6a).

In addition to training and testing with the same type of diffusers, i.e., $L_{train} = L_{test}$, we also tested our single-pixel diffractive network trained with $L_{train} = 25.3\lambda _{\max }$ to classify handwritten digits through $L_{test}$ = 3.2 $\lambda _{\max }$, 10.9 $\lambda _{\max }$, 15.1 $\lambda _{\max }$, 33.8 $\lambda _{\max }$, and 62.3 $\lambda _{\max }$ random diffusers. As shown in Supplementary Fig. S2, the classification accuracy is reduced when $L_{test} \,<\, L_{train}$ and gradually improves when $L_{test} \,>\, L_{train}$, which is eventually upper-bounded by $Acc_0$ = 96.29%. In contrast, a single-pixel diffractive network that was trained without any diffusers, i.e., classifying undistorted handwritten digits, is unable to reject the distortions of a random new diffuser, only achieving $Acc_{new}$ = 33.48% (see Supplementary Fig. S3) when there is a random diffuser presented during the testing stage ($L_{test} = 25.3\lambda _{\max }$). These results further strengthen our conclusions that the diffractive network design converged to a single-pixel handwritten digit classifier with resilience against distortions generated by random unknown diffusers. It also confirms the trade-off between the single-pixel diffractive network’s image distortion resilience and image classification performance/accuracy.

### Experimental demonstration of object classification through an unknown diffuser using a broadband single-pixel diffractive network

**Fig. 7:**
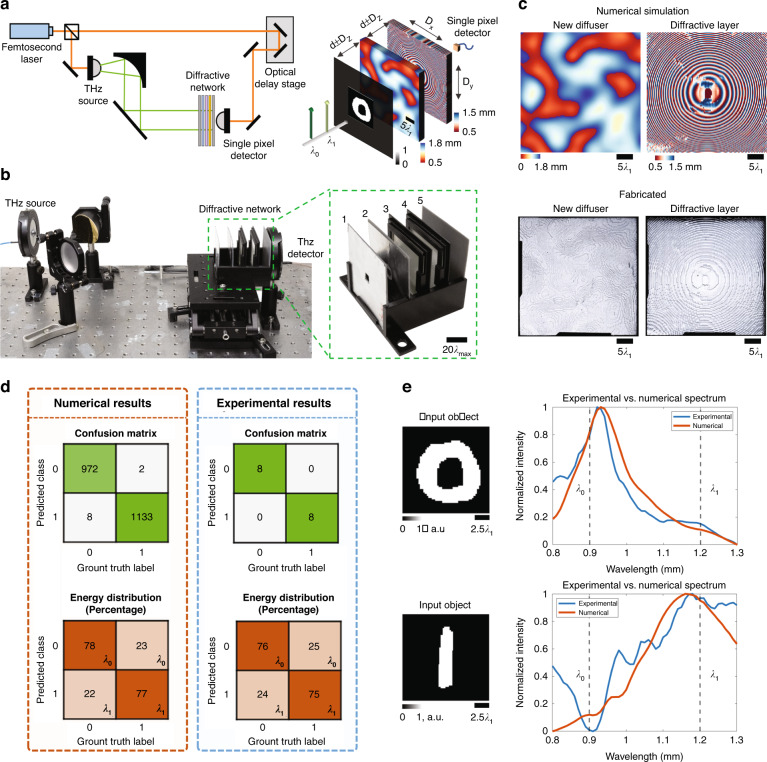
Experimental demonstration of all-optical image classification through an unknown random diffuser using a single-pixel diffractive network. **a** Schematic of the THz-TDS setup and the single-pixel diffractive network trained to all-optically classify handwritten digits “0” and “1” through unknown random phase diffusers. **b** Photograph of the experimental setup and the 3D-printed diffractive network. 1: input aperture; 2: test object; 3: unknown phase diffuser; 4: diffractive layer; 5: single-pixel output aperture. **c** Top: Height profile of the new random diffuser and the trained diffractive layer. Bottom: Fabricated new random diffuser and the diffractive layer used in the experiment. **d** Confusion matrix and the energy distribution percentage for both the numerical simulation and experimental results. **e** Experimental (blue line) and numerical (orange line) output power spectrum for the all-optical classification of handwritten “0” and “1” through a random unknown diffuser, shown as examples

The presented broadband single-pixel classification network was experimentally demonstrated using a THz-TDS system with a 3D-printed diffractive layer and a random new diffuser (Fig. 7a, b). As a proof-of-concept model, we selected handwritten digits “0” and “1” from the MNIST database as two classes to be all-optically classified through random new diffusers. A single-pixel diffractive network with a single diffractive layer, which consists of 120 × 120 diffractive neurons with a neuron size of 0.4 mm, was trained to classify the input objects through randomly generated diffusers using two wavelengths $\lambda _0 =$ 0.9 mm and $\lambda _1 =$ 1.2 mm. The classification result was decoded using a differential scheme as before, based on the relative output power levels of these two wavelengths measured using a single-pixel detector: the input object is classified as “0” when the intensity of $\lambda _0$ is higher than $\lambda _1$, and classified as “1” otherwise. The diffractive network was trained with ***n*** = 80 and ***N*** = 4000 different random diffusers, each with a correlation length of ~25 $\lambda _1$. To accommodate the mechanical misalignments during the system fabrication and assembly, we deliberately introduced random displacements to the diffractive layers to “vaccinate” the diffractive network during the training process^53^ (see Fig. 7a and the “Methods” section for details). The resulting diffractive network with a single layer achieved an average blind testing accuracy of 99.53%, classifying handwritten test digits “0” and “1” through new/unseen random diffusers that were never used in the training stage.

Next, we fabricated the converged diffractive layer using a 3D printer and assembled the diffractive network, as shown in Fig. 7b, c. We also randomly selected and 3D-printed an unknown/new diffuser to experimentally test the fabricated system (see Fig. 7c). The confusion matrix of the numerical blind testing using this selected new diffuser is shown in Fig. 7d. We also calculated the relative energy levels of $\lambda _0$ and $\lambda _1$, as shown in Fig. 7d; these results indicate that the single-pixel diffractive network can classify these two digits with high confidence through a random unknown diffuser. From the correctly classified samples during the numerical blind testing, 16 MNIST handwritten digits (eight “0” and eight “1”) were randomly selected and 3D-printed to form the experimental test objects. The confusion matrix and the energy distribution of the experimental blind testing results using the selected random diffuser are reported in Fig. 7d, where an experimental classification accuracy of 100% was achieved using the fabricated diffractive network to classify the handwritten digits through an unknown new diffuser using a single-pixel THz detector. A good match between the numerically simulated and experimentally measured output spectra and energy distribution can be seen in Fig. 7d, e, which confirms the feasibility of our single-pixel diffractive network in classifying input images all-optically through an unknown random diffuser.

## Discussion

Apart from the deep learning-based training strategies we employed, two design features played important roles in achieving high object-classification accuracies through random, unknown diffusers: (1) the differential spectral encoding scheme and (2) the use of broadband illumination. Optoelectronic detectors can only detect nonnegative optical intensity information, limiting the range of realizable output values of a diffractive network. The differential encoding scheme has been originally proposed to mitigate this constraint by virtually assigning a negative sign to some of the output detectors^29^, which was beneficial for various applications of diffractive networks^31,50,54^. To further reveal the importance of our differential spectral detection scheme (Eq. 1) used for image classification through random diffusers, we trained another diffractive network without any differential encoding using the same physical configuration: the size of the diffractive layers, their spatial arrangement, and the training strategy were kept the same as described in Fig. 2, except that we reduced the number of wavelengths to 10 and used their intensity to directly encode the class labels as opposed to the differential scheme used in Eq. 1. This converged diffractive network scored an accuracy 75.15 ± 8.83% in classifying handwritten digits through new random diffusers, which is on average 13.38% lower than the differentially encoded single-pixel diffractive network’s performance.

Using broadband illumination with a single-pixel detector also enables compactness and simplification of the diffractive system without losing the computational capacity to classify objects accurately through unknown random diffusers. To shed more light on this, we explored and compared other detection schemes using different numbers of wavelengths and spatially distributed output detectors to perform the same classification tasks. To ensure a fair comparison among these different schemes, we kept the degrees of freedom of each detection method the same, i.e., (*Number of detectors*) × (*Number of wavelengths*) = 20 for all the compared cases. More specifically, we employed five different schemes to classify handwritten digits through ~25 $\lambda _{\max }$ random unknown diffusers and trained five diffractive network models for each scheme, following the same training strategy as demonstrated in Fig. 2. These schemes include using 20 wavelengths with a single-pixel detector (the already reported method), 10 illumination wavelengths collected by two spatially separated detectors, five illumination wavelengths with four distinct detectors, two wavelengths with 10 detectors, and a single illumination wavelength with 20 distinct detectors. The physical arrangement of each detection scheme is illustrated in Fig. 2 and Supplementary Figs. S4–S7. For each one of these new detection schemes, the illumination wavelengths were randomly sampled from the 20 wavelengths used in our single-pixel detection scheme (i.e., $\lambda _0,\lambda _1, \ldots ,\lambda _{19}$ in Fig. 1). We repeated the training/blind testing processes five times to obtain the average and standard deviation values of the corresponding classification accuracies through memorized, forgotten, new/unseen, and no diffusers. The confusion matrices reported in Supplementary Figs. S4–S7 and the accuracy plots in Supplementary Fig. S8 demonstrate that there is statistically no significant difference in the classification accuracies for these different configurations. Our broadband diffractive design with a single-pixel detector and 20 wavelengths provides equally competitive classification accuracy through unknown random diffusers despite the fact that the modulation functions of the dielectric diffractive layers at different wavelengths are tightly coupled to each other through the material dispersion. Moreover, our single-pixel diffractive network is advantageous in terms of its simplicity and detection speed without requiring mechanical scanning or a detector array at the output field-of-view. Such a single-pixel diffractive network design is widely useful for applications that involve, for example, using waveguides or fiber-optics for probing hard-to-reach objects and might find applications in spectroscopic analysis and endoscopy.

Although the diffractive networks analyzed in this work used 20 discrete wavelengths, this does not set an upper limit to the number of wavelengths that can be used or object classes that can be classified using broadband diffractive networks. For example, Li et al. reported diffractive networks classifying handwritten letters (composed of 26 classes) using 52 discrete wavelengths with a differential encoding scheme and without any diffusers present^51^. Moreover, earlier work demonstrated the capacity of diffractive networks to process optical waves over a continuous range of frequencies^50^. Recent analyses have further investigated the information processing capacity of a broadband diffractive network^55^ to show that a phase-only diffractive network can approximate $N_w$ unique complex-valued linear transformations using $N_w$ spectral channels (e.g., >180) and $N$ diffractive neurons/features, as long as $N \ge 2N_wN_iN_o$, where $N_i$ and $N_o$ refer to the independent number of pixels at the input and output fields-of-view of the network, respectively.

The random diffuser generation function and the object-to-diffuser distances used in this work were adopted from existing literature to benchmark our system^18,26^. Nevertheless, our approach provides a generic solution to all-optical object classification through unknown new diffusers, where the hyperparameters and diffuser features can be tuned to fit various applications. To exemplify such a scenario, we further tested our single-pixel diffractive model reported in Fig. 2, which was trained with a fixed object-to-diffuser distance (i.e., 33 $\lambda _{\max }$) using different object-to-diffuser distances within a range of 33 $\lambda _{\max }$ ± 4 $\lambda _{\max }$, to classify handwritten digits through unknown random diffusers never seen before. As shown in Supplementary Fig. S9, there is only a small amount of degradation in the blind classification accuracy when the object-to-diffuser distance changes with respect to its training value (see the green curve in Supplementary Fig. S9b). This relatively small degradation in classification performance can be mitigated by intentionally introducing random displacements of the objects/diffusers during the training stage. To demonstrate this, we trained, from scratch, another three-layer “vaccinated” single-pixel diffractive network in which the axial positions of the objects were randomly shifted by an amount of ${{{\mathrm{{\Delta}}}}}z$, randomly sampled from a uniform distribution ${{{\mathbf{U}}}}( - 4\lambda _{\max },4\lambda _{\max })$. Such a “vaccination” training strategy largely enhanced the robustness of our single-pixel diffractive network to varying object-to-diffuser distances, where the classification accuracy remained largely unchanged when the trained model was tested with varying object-to-diffuser distances (see the blue curve in Supplementary Fig. S9).

Besides object-to-diffuser distance variations, we also trained and tested two new three-layer single-pixel diffractive network models with different layer-to-layer distances (4.2 $\lambda _{\max }$ and 33.3 $\lambda _{\max }$) to perform the same classification task through random new diffusers with a correlation length of ~25 $\lambda _{\max }$, while keeping the object-to-diffuser distance at 33 $\lambda _{\max }$. As shown in Supplementary Fig. S10, a similar level of image classification accuracy through new random diffusers was observed for these models. These results further highlight the flexibility of our single-pixel diffractive network as a generic solution framework adaptable to different configurations and design parameters.

We should also note that the presented single-pixel diffractive network is not limited to the all-optical classification of objects distorted by random phase diffusers with Gaussian statistics. To shed more light on this, we extended the diffuser definitions used in our work and trained two additional single-pixel diffractive networks for classifying handwritten digits through random (1) linear and (2) circular gratings that are never seen by the network before (see the “Methods” section for details). Example images of objects seen through such grating-like diffusers are shown in Supplementary Fig. S11, highlighting the impact of such phase diffusers on hiding/distorting the information of the input objects. After the training of the single-pixel diffractive networks with these two types of grating-like diffusers, the handwritten digit classification results through random new diffusers are summarized in Supplementary Figs. S12–S13, achieving $Acc_{new} = 94.82\%$ and $Acc_{new} = 94.08\%$ for the linear and circular phase diffusers, respectively. It is worth noting that $Acc_m \approx Acc_f \approx Acc_{new} > Acc_0$ when the single-pixel diffractive network was trained with these grating-like random diffusers. This indicates that the single-pixel network can very well generalize to classify input objects seen through such linear or circular random gratings since they carry less diverse spatial features, helping us achieve $Acc_m \approx Acc_f \approx Acc_{new}$.

Compared to performing all-optical reconstruction of input objects distorted by unknown diffusers, as demonstrated in our former work^26^, the presented framework of all-optical classification through unknown diffusers tackles a more challenging task with an additional level of complexity as it requires the ability to assign the input objects to the correct classes using a single-pixel diffractive network. In contrast, our former work^26^ projected a reconstructed image at its output plane that needs to be digitized and stored by the pixels of a focal plane array or CMOS imager before a machine learning algorithm (for example, a digital neural network) can classify the reconstructed and digitized/stored image. The presented diffractive network that classifies objects through random unknown diffusers is based on a single pixel, and it directly performs all-optical classification of the input objects without an image reconstruction step or digitization by a focal plane array or the use of a digital image classification network. Such a single-pixel diffractive network design can be widely useful for applications that involve, for example, a single waveguide or fiber-optic cable for probing hard-to-reach objects. This framework can find applications in various fields where the image reconstruction alone does not provide a complete solution and continuous/automated object recognition behind random diffusers is required, such as in security surveillance and autonomous driving.

In conclusion, we presented an all-optical processer to classify unknown objects through random, unknown diffusers using a broadband single-pixel diffractive network. Designed to classify handwritten digits through random unknown diffusers, the single-pixel broadband diffractive network memorized the diffusers used in the last training epoch, scoring $Acc_m$ = 91.98 ± 0.24% image classification accuracy when the random diffusers from the last training epoch were used. The same single-pixel broadband diffractive network can also classify blind objects through unknown new diffusers never used in training, achieving an average accuracy of $Acc_{new}$ = 87.74 ± 1.12%. This diffractive framework was also applied to classify objects through random phase diffusers with various correlation lengths showing an improved classification accuracy when random diffusers with larger correlation lengths were used since the input images were less distorted. Our experiments further confirmed the applicability of our single-pixel broadband diffractive network for all-optical image classification through an unknown random diffuser, demonstrating the feasibility of the presented approach. The image classification through random diffusers requires no external computing power except for the illumination source, presenting a time- and energy-efficient solution. The teachings of this all-optical processer will find unique applications in many fields, such as surveillance cameras, security, biomedical imaging, and autonomous driving.

## Materials and methods

### Model of a broadband single-pixel diffractive network

Broadband illumination was used for object classification through unknown diffusers. The diffractive layers were modeled as thin optical modulation elements, where the $i^{th}$ neuron on the $l^{th}$ layer at a spatial location ($x_i,y_i,z_i$) represents a wavelength ($\lambda$)-dependent complex-valued transmission coefficient, $t^l$, given by:

2 $$\begin{array}{*{20}{c}} {t^l\left( {x_i,y_i,z_i,\lambda } \right) = a^l\left( {x_i,y_i,z_i,\lambda } \right)\exp \left( {j\phi ^l\left( {x_i,y_i,z_i,\lambda } \right)} \right)} \end{array}$$

In this work, we assumed $a^l\left( {x_i,y_i,z_i,\lambda } \right) = 1$. The phase modulation $\phi ^l\left( {x_i,y_i,z_i,\lambda } \right)$ can be written as a function of the thickness of each diffractive neuron $h_i^l$ and the incident wavelength $\lambda$:

3 $$\begin{array}{*{20}{c}} {\phi ^l\left( {x_i,y_i,z_i,\lambda } \right) = \left( {n\left( \lambda \right) - n_{air}} \right)\frac{{2\pi h_i^l}}{\lambda }} \end{array}$$

where $n\left( \lambda \right)$ is the refractive index of the diffractive material. In this work, the height of each neuron was defined as:

4 $$\begin{array}{*{20}{c}} {h_i^l = \frac{{h_{\max }}}{2} \cdot \left( {\sin \left( {h_p} \right) + 1} \right) + h_{base}} \end{array}$$

where $h_p$ is the latent variable that was optimized during the data-driven training procedure. The ultimate height of each diffractive neuron $h_i^l$ was constrained by setting $h_{\max }$ = 0.83 $\lambda _{\max }$, with a fixed base height $h_{base}$ = 0.42 $\lambda _{\max }$. The diffractive layers were optically connected to each other by diffracted light propagation in free space, which was modeled through the Rayleigh–Sommerfeld diffraction equation^28,50^. Each neuron ($x_i,y_i,z_i$) on $l^{th}$ layer can be viewed as a secondary wave source, generating a complex-valued field $w_i^l\left( {x,y,z,\lambda } \right)$ at a spatial location of $\left( {x,y,z} \right)$, which can be formulated as:

5 $$\begin{array}{*{20}{c}} {w_i^l\left( {x,y,z,\lambda } \right) = \frac{{z - z_i}}{{r^2}}\left( {\frac{1}{{2\pi r}} + \frac{1}{{j\lambda }}} \right)\exp \left( {\frac{{j2\pi r}}{\lambda }} \right)} \end{array}$$

where $r = \sqrt {(x - x_i)^2 + (y - y_i)^2 + (z - z_i)^2}$ and $j = \sqrt { - 1}$. For the $l^{th}$ layer ($l \ge$1), the modulated optical field $u^l$ at location ($x_i,y_i,z_i$) is given by:

6 $$\begin{array}{l} u^l\left( {x_i,y_i,z_i,\lambda } \right) = t^l\left( {x_i,y_i,z_i,\lambda } \right) \\ \qquad\qquad\qquad\quad\cdot \mathop {\sum}\limits_{k \in I} {u^{l - 1}\left( {x_k,y_k,z_k,\lambda } \right) \cdot w_k^{l - 1}\left( {x_i,y_i,z_i,\lambda } \right)} \end{array}$$

where $I$ denotes all the diffractive neurons on the previous (i.e., $l - 1^{th}$) diffractive layer. In case of $l$ = 1, $u^0\left( {x_k,y_k,z_k,\lambda } \right)$ denotes the optical field right after the random diffuser, which can be formulated as:

7 $$\begin{array}{l} {u^0\left( {x_i,y_i,z_i,\lambda } \right) = t^D\left( {x_i,y_i,z_i,\lambda } \right) \cdot \mathop {\sum}\limits_{k \in I} {o\left( {x_k,y_k,z_k,\lambda } \right) \cdot w_k^{OD}\left( {x_i,y_i,z_i,\lambda } \right)} } \end{array}$$

where $o\left( {x_k,y_k,z_k,\lambda } \right)$ is the transmission function of a pixel at the input object plane and $w_k^{OD}$ denotes the free-space propagation from the object plane to the diffuser plane. $t^D\left( {x_i,y_i,z_i,\lambda } \right)$ is the modulation generated by a random phase diffuser, which can be calculated using its height map $h_D$ and Eq. (3). The height map of each random phase diffuser was defined as:

8 $$\begin{array}{*{20}{c}} {h_D\left( {x,y} \right) = rem\left( {W\left( {x,y} \right) \ast K\left( \sigma \right) + h_{base},\frac{{\lambda _{\max }}}{{n\left( {\lambda _{\max }} \right) - n_{air}}}} \right)} \end{array}$$

where $rem\left( . \right)$ denotes the remainder after division. $W\left( {x,y} \right)$ is a random height matrix that follows a normal distribution with a mean of *μ* and a standard deviation of $\sigma _0$, i.e.,

9 $$\begin{array}{*{20}{c}} {W\left( {x,y} \right)\sim {{{\mathcal{N}}}}\left( {\mu ,\sigma _0} \right)} \end{array}$$

$K\left( \sigma \right)$ is a Gaussian smoothing kernel with zero mean and standard deviation $\sigma$. “*” denotes the 2D convolution operation. The correlation length ($L$) of a random diffuser was calculated using the 2D auto-correlation function $(R_d)$ of its height profile $h_D\left( {x,y} \right)$, based on the following equation:

10 $$R_d\left( {x,y} \right) = \exp \left( { - \pi \left( {x^2 + y^2} \right)/L^2} \right)$$

A single-pixel detector was placed on the optical axis at the end of the diffractive network, after the $L^{th}$ layer, which measured the intensity at each encoding wavelength within a square aperture of 3 $\lambda _{\max }$ by 3 $\lambda _{\max }$ The single-pixel spectral measurement $s_p$ at a wavelength of $\lambda _p$ can be formulated as:

11 $$\begin{array}{*{20}{c}} {s_p = \left| {\mathop {\sum}\limits_{k \in I} {u^L\left( {x_k,y_k,z_k,\lambda _p} \right) \cdot w_k^L\left( {x_i,y_i,z_i,\lambda _p} \right)} } \right|^2} \end{array}$$

The differential spectral class scores were calculated following Eq. (1), and the diffractive networks were trained to optimize the classification accuracy using a softmax cross-entropy (SCE) loss:

12 $$\begin{array}{*{20}{c}} {{{{\mathcal{L}}}}_I = - \mathop {\sum}\limits_{c = 0}^9 {g_c \cdot {{{\mathrm{log}}}}\left( {\frac{{\exp \left( {{\Delta}s_c} \right)}}{{\mathop {\sum}\nolimits_{c\prime = 0}^9 {\exp \left( {{\Delta}s_{c\prime }} \right)} }}} \right)} } \end{array}$$

where ${\Delta}s_c$ denotes the spectral class score for the $c^{{{{\mathrm{th}}}}}$ class, and $g_c$ denotes the $c^{{{{\mathrm{th}}}}}$ entry of the ground truth label vector.

The height map of each random linear grating diffuser was defined as:

13 $$\begin{array}{*{20}{c}} {h_{D - linear}(x,y) = \frac{{\lambda _{\max }}}{{2\left( {n\left( {\lambda _{\max }} \right) - n_{air}} \right)}} \cdot \sin \left( {2\pi \frac{{x\cos \left( \theta \right) + y\sin \left( \theta \right)}}{{a_0}}} \right) + h_{base}} \end{array}$$

where $a_0$ is the period of the linear grating and was randomly sampled from the uniform distribution ${{{\mathbf{U}}}}(12\lambda _{\max },16\lambda _{\max })$ during the random grating generation. $\theta$ controls the direction of the grating, which is also a random variable following the uniform distribution ${{{\mathbf{U}}}}(0,2\pi )$. Similarly, the height map of each random circular grating diffuser was defined as:

14 $$\begin{array}{*{20}{c}} {h_{D - circular}(x,y) = \frac{{\lambda _{\max }}}{{2(n\left( {\lambda _{\max }} \right) - n_{air})}} \cdot \sin \left( {2\pi \frac{{\sqrt {\left( {x - x_0} \right)^2 + \left( {y - y_0} \right)^2} }}{{a_0}}} \right) + h_{base}} \end{array}$$

where $(x_0,y_0)$ is the coordinate of the center of the circular grating. $x_0$ and $y_0$ were independently sampled from the uniform distribution ${{{\mathbf{U}}}}( - 37.5\lambda _{\max },37.5\lambda _{\max })$. $a_0$ is the period of the circular grating, randomly sampled from the uniform distribution ${{{\mathbf{U}}}}(12\lambda _{\max },16\lambda _{\max })$.

### Digital implementation

The diffractive neural networks presented here contained 200 × 200 diffractive neurons on each layer with a pixel size (pitch) of 0.25 $\lambda _{\max }$. During the training, each handwritten digit of the MNIST dataset was first upscaled from 28 × 28 pixels to 70 × 70 pixels using bilinear interpolation and then padded with zeros to cover 200 × 200 pixels. The broadband illumination was digitally modeled as multiple independently propagating monochrome plane waves; we used $\lambda _{\min } = 0.6\,{{{\mathrm{mm}}}}$ and $\lambda _{\max } = 1.2\,{{{\mathrm{mm}}}}$ based on the THz part of the spectrum. The propagation and wave modulation on each spectral channel were separately computed. Four different randomly selected MNIST images formed a training batch, providing amplitude-only modulation to the input broadband light. Each input object batch was propagated and disturbed by one randomly selected diffuser. The four distorted broadband fields were separately propagated through the diffractive network, and the loss value (Eq. (12)) was calculated accordingly. The resulting loss was back-propagated, and the pixel height values were updated using the Adam optimizer^56^ with a learning rate of $1 \times 10^{ - 3}$. Our models were trained using Python (v3.7.3) and PyTorch (v1.11) for 100 epochs, which took 5 h to complete. A desktop computer with a GeForce RTX 3090 graphical processing unit (GPU, Nvidia Inc.), an Intel® Core ™ i9-7900X central processing unit (CPU, Intel Inc.), and 64 GB of RAM was used.

### Experimental design and THz-TDS system

The diffuser and the diffractive layer used for the experimental demonstration were fabricated using a 3D printer (Pr 110, CADworks3D). The 3D printing material we used in the experiments has wavelength-dependent absorption. Therefore, additional neuron height-dependent amplitude modulations were applied to the incident light, which can be formulated as

15 $$\begin{array}{*{20}{c}} {a^l\left( {x_i,y_i,z_i,\lambda } \right) = \exp \left( { - \frac{{2\pi \kappa \left( \lambda \right)h_i^l}}{\lambda }} \right)} \end{array}$$

where $\kappa \left( \lambda \right)$ is the extinction coefficient of the diffractive layer material, corresponding to the imaginary part of the complex-valued refractive index $\tilde n\left( \lambda \right)$, i.e., $\tilde n\left( \lambda \right) = n\left( \lambda \right) + j\kappa \left( \lambda \right)$.

For the single-layer single-pixel diffractive model used for the experimental demonstration (Fig. 7), the diffractive layer consists of 120 × 120 diffractive neurons, each with a lateral size of 0.4 mm. The axial separation between any two consecutive planes was set to *d* = 20 mm. To compensate for the nonideal wavefront generated by the THz emitter, a square input aperture with a size of 8 × 8 mm^2^ was used as an entrance pupil to illuminate the input object, placed 20 mm away from it. The diffraction of this aperture was also included in the forward propagation model. The size of the input objects was designed as 20 × 20 mm^2^ (50 × 50 pixels). After being distorted by the random diffuser and modulated by the diffractive layer, the spectral power at the center region (2.4 × 2.4 mm^2^) of the output plane was measured to determine the class score.

To overcome potential mechanical misalignments during the experimental testing, the network was “vaccinated” with deliberate random displacements during the training stage^53^. Specifically, a random lateral displacement $\left( {D_x,D_y} \right)$ was added to the diffractive layer, where $D_x$ and $D_y$ were randomly and independently sampled, i.e.,

16 $$\begin{array}{*{20}{c}} {D_x\sim {\mathbf{U}}\left( { - 0.4\,{{{\mathrm{mm}}}},0.4\,{{{\mathrm{mm}}}}} \right),D_y\sim {\mathbf{U}}\left( { - 0.4\,{{{\mathrm{mm}}}},0.4\,{{{\mathrm{mm}}}}} \right)} \end{array}$$

where $D_x$ and $D_y$ are not necessarily equal to each other in each misalignment step.

A random axial displacement $D_z$ was also added to the axial separations between any two consecutive planes. Accordingly, the axial distance between any two consecutive planes was set to $d \pm D_z =$ 20 mm $\pm D_z$, where $D_z$ was randomly sampled as,

17 $$\begin{array}{*{20}{c}} {D_z\sim {{{\mathbf{U}}}}\left( { - 0.2\,{{{\mathrm{mm}}}},0.2\,{{{\mathrm{mm}}}}} \right)} \end{array}$$

In our experiments, we also measured the power spectrum of the pulsed terahertz source with only the input and output apertures present, which served as an experimental reference spectrum, $I_{ref}(\lambda )$. Based on this, the experimentally measured power spectrum at the output single-pixel aperture of a diffractive network can be written as:

18 $$\begin{array}{*{20}{c}} {s_{i,calibrated} = \frac{{s_{i,measured}}}{{I_{ref}\left( {\lambda _i} \right)}}} \end{array}$$

The binary objects and apertures were all 3D-printed (Form 3B, Formlabs) and coated with aluminum foil to define the transmission areas. Apertures, objects, the diffuser, and the diffractive layer were assembled using a 3D-printed holder (Objet30 Pro, Stratasys). The setup of the THz-TDS system is illustrated in Fig. 7a. A Ti:Sapphire laser (Mira-HP, Coherent) generates optical pulses with a 135-fs pulse width and a 76-MHz repetition rate at a center wavelength of 800 nm, which pumps both a high-power plasmonic photoconductive terahertz source^57^ and a high-sensitivity plasmonic photoconductive terahertz detector^58^. The terahertz radiation generated by the terahertz source is collimated by a 90° off-axis parabolic mirror and illuminates the test object. After interacting with the object, the diffuser, and the diffractive neural network, the radiation is coherently detected by the terahertz detector (single-pixel). A transimpedance amplifier (DHPCA-100, Femto) converts the current signal to a voltage signal, which is then measured by a lock-in amplifier (MFLI, Zurich Instruments). By varying the optical delay between the terahertz radiation and the optical probe beam on the terahertz detector, the terahertz time-domain signal can be obtained. By taking the Fourier transform of the time-domain signal, the spectral intensity signal is revealed to calculate the class scores for each classification/inference. For each measurement, 10 time-domain traces are collected and averaged. This THz-TDS system provides a signal-to-noise ratio larger than 90 dB and a detection bandwidth larger than 4 THz.

## Supplementary information

Supplementary Information

## Data Availability

All the data and methods needed to evaluate the conclusions of this work are present in the main text and the Supplementary Material. Additional data can be requested from the corresponding author.

## Code availability

The deep learning models reported in this work used standard libraries and scripts that are publicly available in PyTorch.
