# Supplementary material for: All-optical image classification through unknown random diffusers using a single-pixel diffractive network
Source: Light Sci Appl. 2023 Mar 9;12:69. doi: 10.1038/s41377-023-01116-3 (PMC9998891; doi:10.1038/s41377-023-01116-3)
Supplement: Supplementary file 1 — Supplementary Information [file 41377_2023_1116_MOESM1_ESM.pdf]

Supplementary Information for

All-optical image classification through unknown random diffusers using a single-pixel diffractive network

Bijie Bai[1,2,3,+] e-mail: baibijie@g.ucla.edu

Yuhang Li[1,2,3,+] e-mail: yuhangli@g.ucla.edu

Yi Luo[1,2,3,+] e-mail: yluo2016@ucla.edu

Xurong Li[1,3] e-mail: xurongli@ucla.edu

Ege Çetintaş[1,2,3] e-mail: egecetintas1@g.ucla.edu

Mona Jarrahi[1,3] e-mail: mjarrahi@ucla.edu

Aydogan Ozcan[1,2,3*] e-mail: ozcan@ucla.edu

[1]Electrical and Computer Engineering Department, University of California, Los Angeles, California 90095, USA

[2]Bioengineering Department, University of California, Los Angeles, California 90095, USA

[3]California NanoSystems Institute (CNSI), University of California, Los Angeles, California 90095, USA

[+]Equal contributing authors

[*]Correspondence: Prof. Aydogan Ozcan E-mail: ozcan@ucla.edu

Address: 420 Westwood Plaza, Engr. IV 68-119, UCLA, Los Angeles, CA 90095, USA

Tel: +1(310)825-0915

Fax: +1(310)206-4685

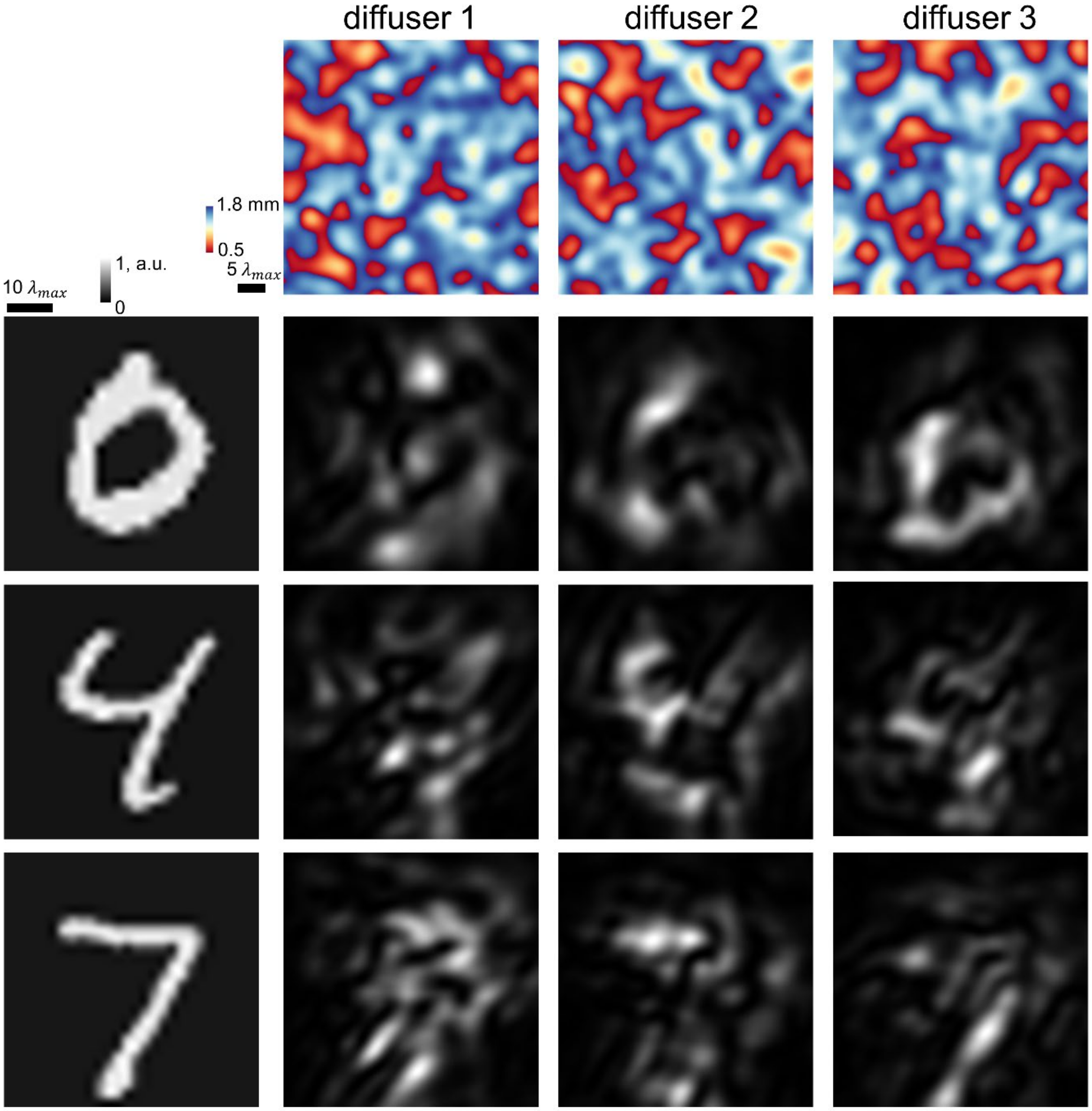

Figure S1. Imaging of test objects with a wavelength of 1.2 mm (λ_{max}) through three different random diffusers, each with a correlation length of 25.3λ_{max} .

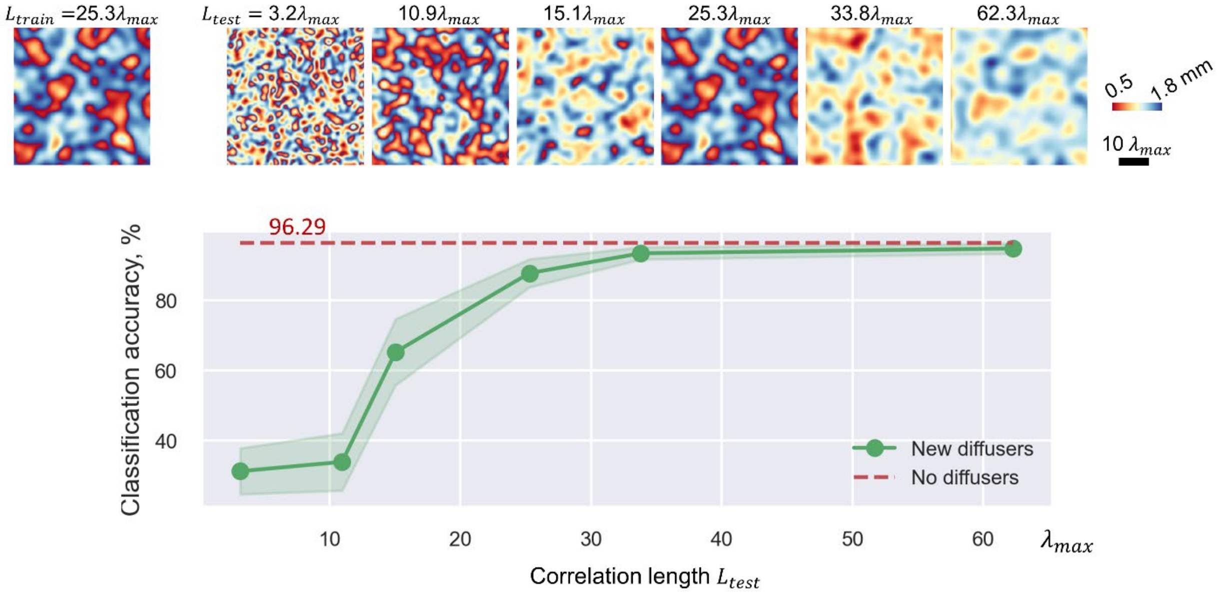

Figure S2. **Classification performance of the single-pixel diffractive network (composed of 3 layers) trained with random diffusers with a correlation length of $L_{train} = 25.3\lambda_{max}$ and tested with random diffusers with various correlation lengths (L_{test}).** Top: Demonstration of random diffusers with different correlation lengths. Bottom: Classification accuracy of the single-pixel diffractive network trained with $L_{train} = 25.3\lambda_{max}$ random diffusers, classifying unknown handwritten digits through random diffusers with different L_{test}. We also show, with a dashed red line, the classification accuracy of the same single-pixel diffractive network trained with $L_{train} = 25.3\lambda_{max}$ random diffusers and tested without any diffusers (achieving a classification accuracy of 96.29%).

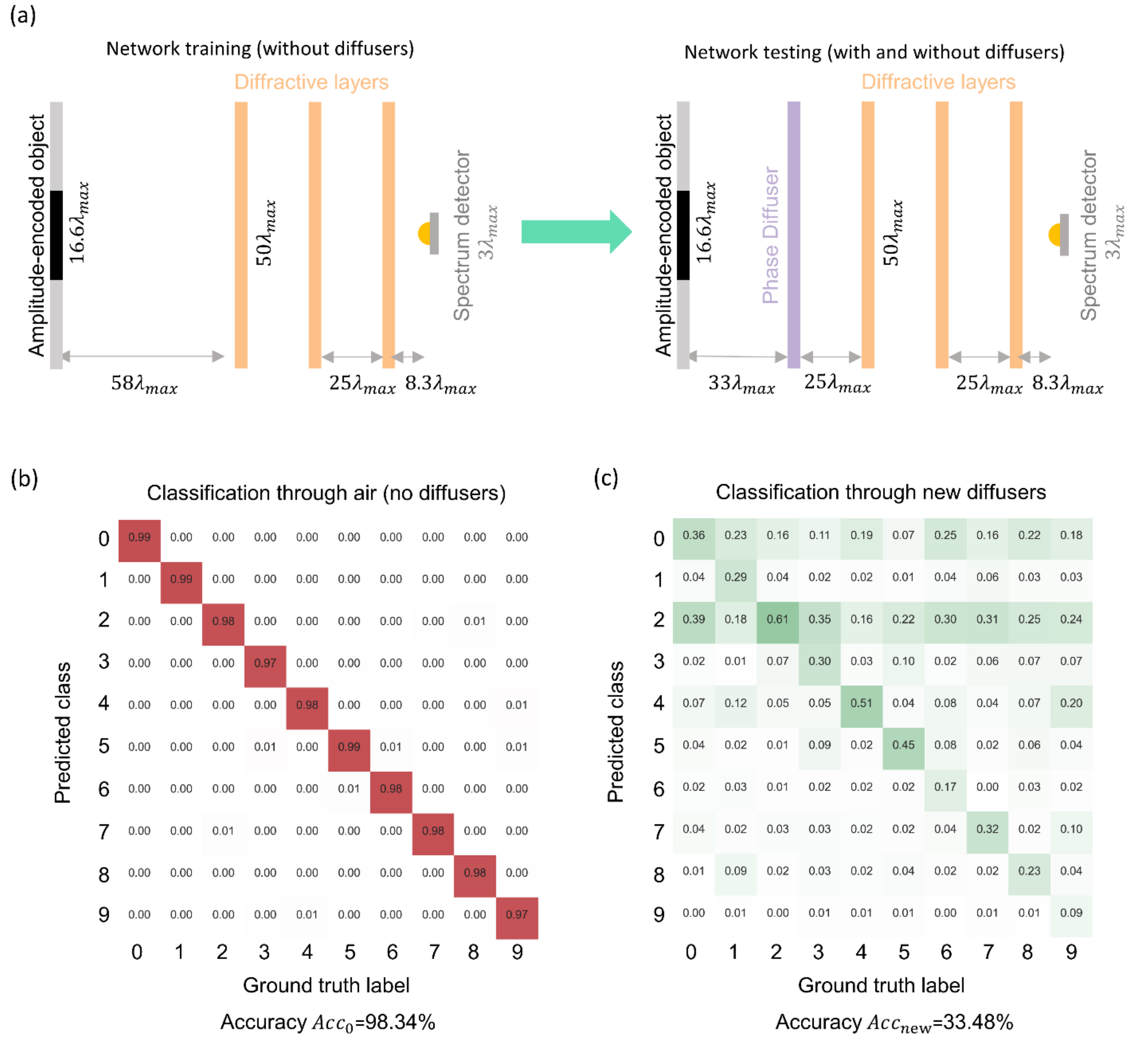

Figure S3. **Classification performance of a single-pixel diffractive network trained without any diffusers and tested with or without diffusers.** (a) The schematic of training without phase diffusers and testing with and without diffusers. (b) Confusion matrix for blind testing without diffusers; $Acc_0 = 98.34\%$. (c) Confusion matrix for blind testing with random diffusers; $Acc_{new} = 33.48\%$.

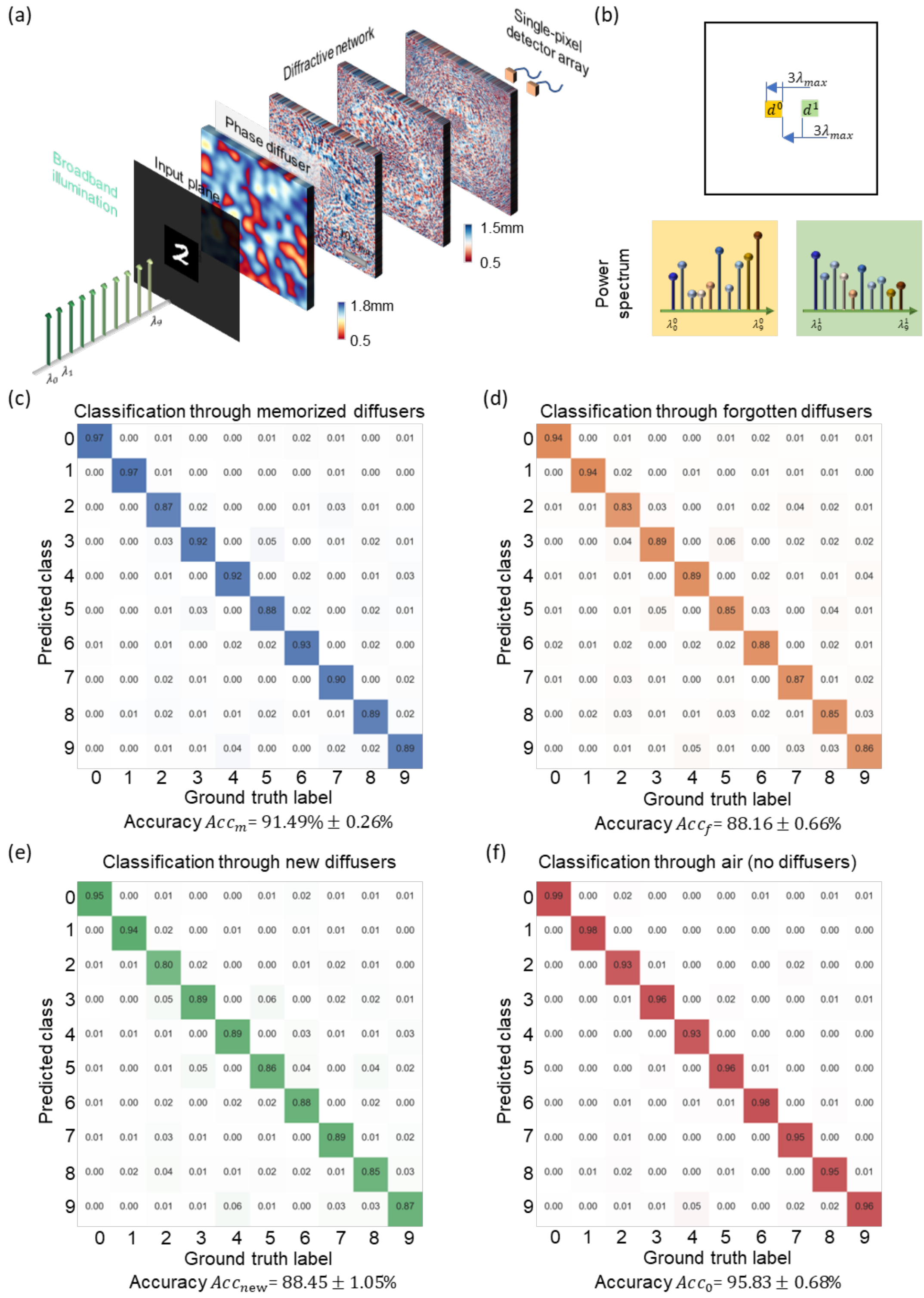

Figure S4. **Broadband diffractive networks with 2 detectors and 10 pre-determined wavelengths to classify unknown objects through random phase diffusers.** (a) The diffractive network designed to classify MNIST handwritten digits placed behind unknown random diffusers using three successive diffractive layers and 10 discrete illumination wavelengths. Five models with the same parameters except

for the wavelengths used were trained. For each model, the illumination wavelengths were randomly selected from 20 wavelengths used in our single-pixel detection scheme ranging from λ_{min} = 0.6 mm to λ_{max} = 1.2 mm, and sorted in ascending order. (b) The spatial information of each handwritten digit placed at the input plane was first encoded into the amplitude channel of all the 10 wavelengths. Two detectors denoted as $\{d^0, d^1\}$ were placed at the output plane and were $3\lambda_{max}$ away from each other. Each of them measures the intensity of all 10 wavelengths. $\{\lambda_0^0, \lambda_1^0, \dots, \lambda_9^0\}$ represents the power spectrum measured by the detector d^0. Similarly, $\{\lambda_0^1, \lambda_1^1, \dots, \lambda_9^1\}$ is the measurement of the detector d^1. The spectral measurements $\{\lambda_0^0, \lambda_1^0, \dots, \lambda_9^0\}$ of d^0 were assigned to $(s_0, s_1, \dots, s_9)$ and the spectral measurements $\{\lambda_0^1, \lambda_1^1, \dots, \lambda_9^1\}$ of d^1 were assigned to $(s_{10}, s_{11}, \dots, s_{19})$. The class scores for each class c were calculated as $output_c = \frac{s_c - s_{c+10}}{s_c + s_{c+10}}$, where the highest output score defines the final classification decision for the input object behind the random diffuser. Confusion matrices for the diffractive network classifying unknown objects through memorized (c), forgotten (d), new (e), and no diffusers (f), respectively. The average and standard deviation values of the classification accuracy were calculated using five different models trained with the same parameters except for the illumination wavelengths.

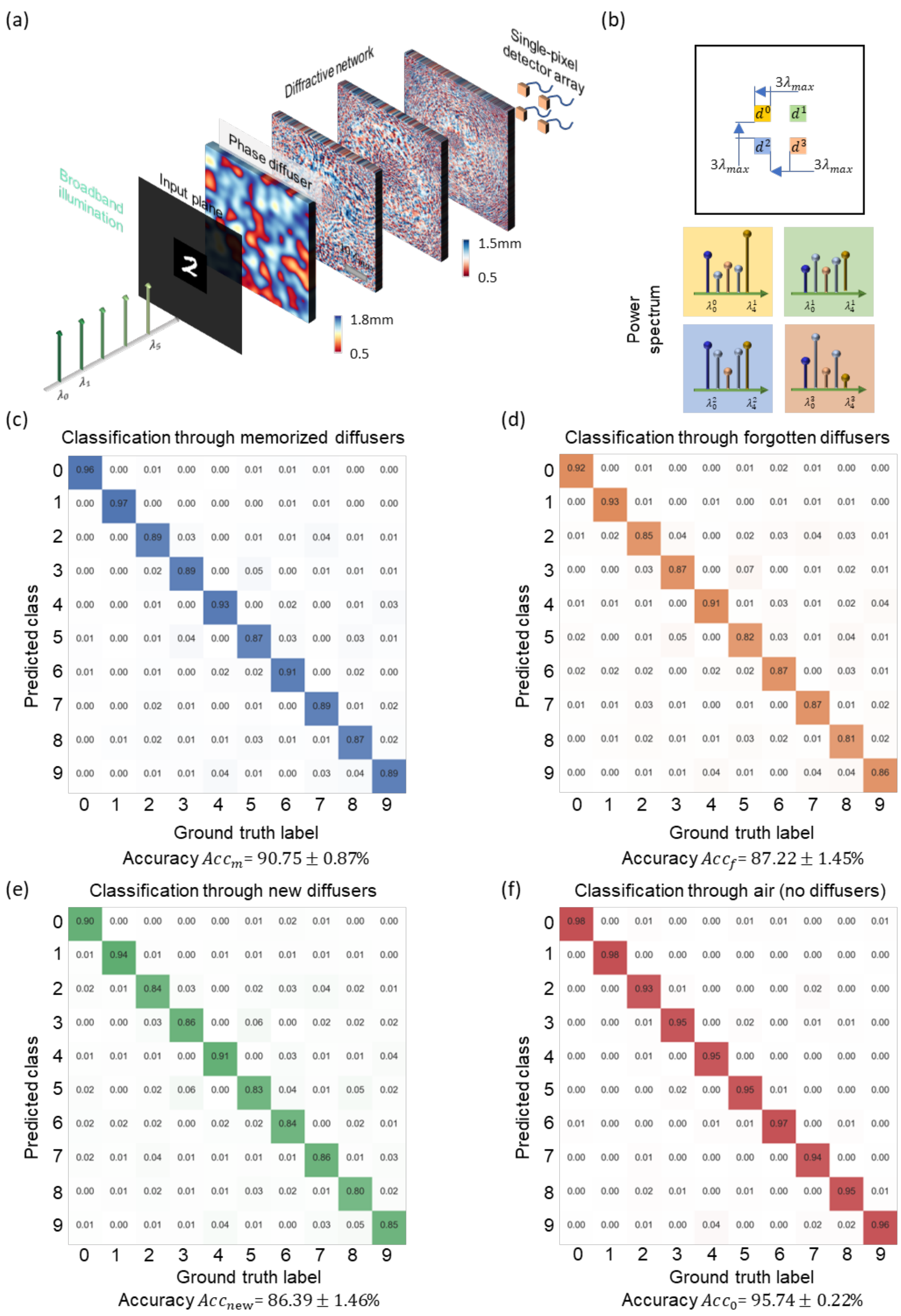

Figure S5. Broadband diffractive networks with 4 detectors and 5 pre-determined wavelengths to classify unknown objects through random phase diffusers. (a) The diffractive network designed to classify MNIST handwritten digits placed behind unknown random diffusers using three successive

diffractive layers and five discrete illumination wavelengths. Five models with the same parameters except for the wavelengths used were trained. For each model, the illumination wavelengths were randomly selected from 20 wavelengths used in our single-pixel detection scheme ranging from λ_{min} = 0.6 mm to λ_{max} = 1.2 mm, and sorted in ascending order. (b) The spatial information of each handwritten digit placed at the input plane was first encoded into the amplitude channel of all the five wavelengths. Four detectors denoted as $\{d^0, d^1, d^2, d^3\}$ were placed at the output plane and were $3\lambda_{max}$ away from each other. Each of them measures the intensity of all five wavelengths. $\{\lambda_0^i, \lambda_1^i, \ldots, \lambda_4^i\}$ represents the power spectrum measured by the detector d^i, where $i \in \{0,1,2,3\}$. The spectral measurements $\{\lambda_0^i, \lambda_1^i, \ldots, \lambda_4^i\}$ of d^i were assigned to $(s_{5i}, s_{5i+1}, \ldots, s_{5i+4})$. The class scores for each class c were calculated as $output_c = \frac{s_c - s_{c+10}}{s_c + s_{c+10}}$, where the highest output score defines the final classification decision for the input object. Confusion matrices for the diffractive network classifying unknown objects through memorized (c), forgotten (d), new (e), and no diffusers (f), respectively. The average and standard deviation values of the classification accuracy were calculated using five different models trained with the same parameters except for the illumination wavelengths.

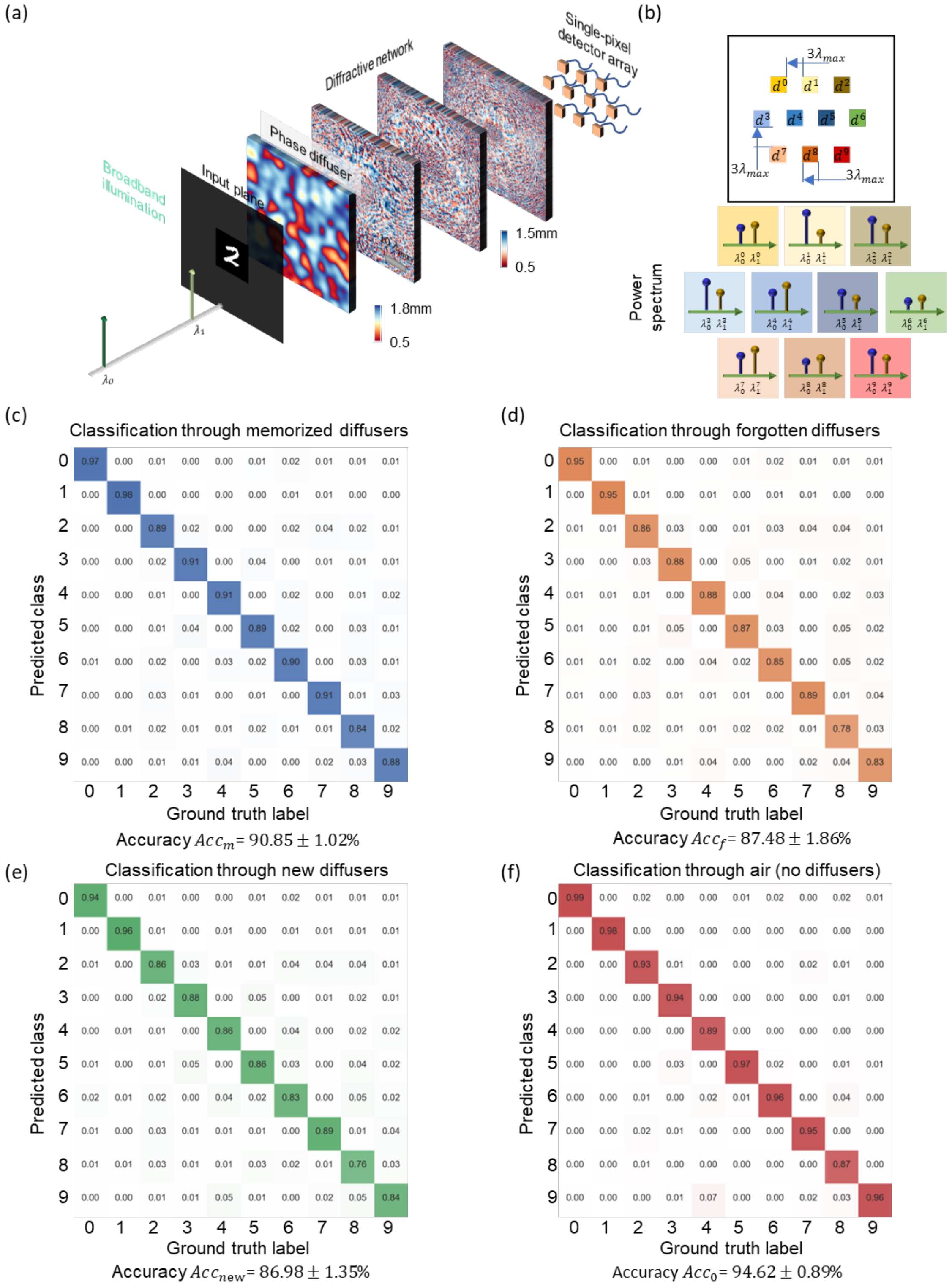

Figure S6. Broadband diffractive networks trained with 10 detectors and 2 pre-determined wavelengths to classify unknown objects through random phase diffusers. (a) The diffractive network designed to classify MNIST handwritten digits placed behind unknown random diffusers using three successive diffractive layers and two discrete illumination wavelengths. Five models with the same

parameters except for the wavelengths used were trained. For each model, the illumination wavelengths were randomly selected from 20 wavelengths used in our single-pixel detection scheme ranging from λ_{min} = 0.6 mm to λ_{max} = 1.2 mm and sorted in ascending order. (b) The spatial information of each handwritten digit placed at the input plane was first encoded into the amplitude channel of the two wavelengths. Ten detectors denoted as $\{d^0, d^1, \dots, d^9\}$ were placed at the output plane and were $3\lambda_{max}$ away from each other. Each of them measures the intensity of two wavelengths. $\{\lambda_0^i,\ \lambda_1^i\}$ represents the power spectrum measured by the detector d^i, where $i \in \{0,1,2, \dots ,9\}$. The spectral measurements $\{\lambda_0^i,\ \lambda_1^i\}$ of d^i were assigned to (s_{2i}, s_{2i+1}). The class scores for each class c were calculated by the differential method as $output_c = \frac{s_c - s_{c+10}}{s_c + s_{c+10}}$, where the highest output score defines the final classification decision for the input object. Confusion matrices for the diffractive network classifying unknown objects through memorized (c), forgotten (d), new (e), and no diffusers (f), respectively. The average and standard deviation values of the classification accuracy were calculated using five different models trained with the same parameters except for the illumination wavelengths.

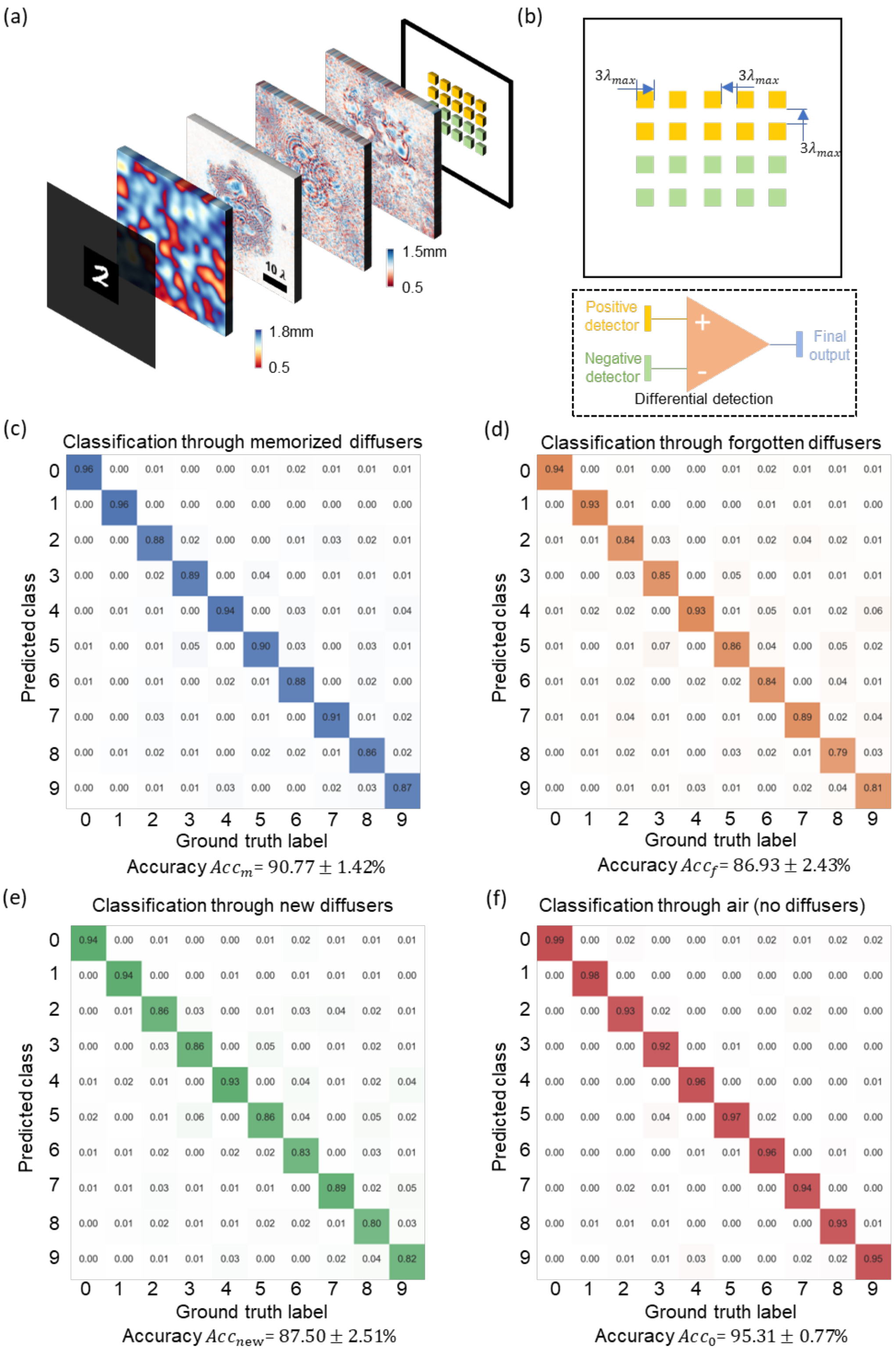

Figure S7. Monochrome diffractive networks trained with 20 detectors to classify MNIST handwritten digits through random phase diffusers. (a) The monochrome diffractive network designed to classify objects placed behind unknown random diffusers using three successive diffractive layers and a single illumination wavelength. Five models with the same parameters except for the illumination

wavelength were trained. The illumination wavelengths were randomly selected from 20 wavelengths used in our single-pixel detection scheme ranging from $\lambda_{min} = 0.6$ mm to $\lambda_{max} = 1.2$ mm to match the range used in the main text. (b) 20 detectors denoted as $\{s_0, s_1, \dots, s_{19}\}$ were placed at the output plane and were $3\lambda_{max}$ away from each other. The upper ten yellow detectors $\{s_0, s_1, \dots, s_9\}$ were denoted as positive detectors and the lower ten green detectors $\{s_{10}, s_{11}, \dots, s_{19}\}$ were used as negative detectors. The class scores for each class c were calculated as $output_c = \frac{s_c - s_{c+10}}{s_c + s_{c+10}}$. Confusion matrices for the diffractive network classifying unknown objects through memorized (c), forgotten (d), new (e), and no diffusers (f), respectively. The average and standard deviation values of the classification accuracy were calculated using five different models trained with the same parameters except for the illumination wavelengths.

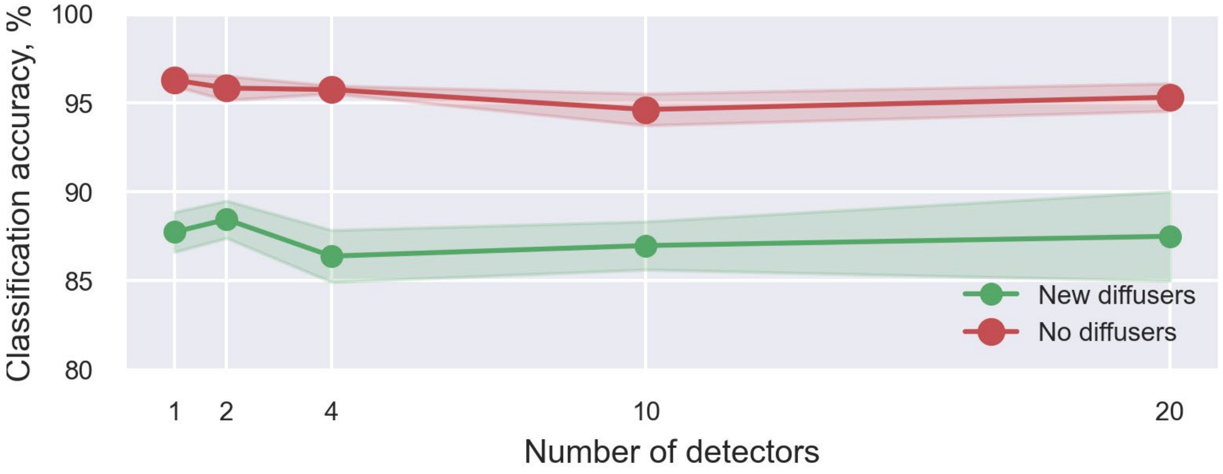

Figure S8. **Classification accuracy of single-pixel diffractive neural networks trained with different combinations of (number of detectors, number of wavelengths)**: (1, 20), (2, 10), (4, 5), (10, 2), (20, 1) through new and no diffusers, respectively.

(a)

(b)

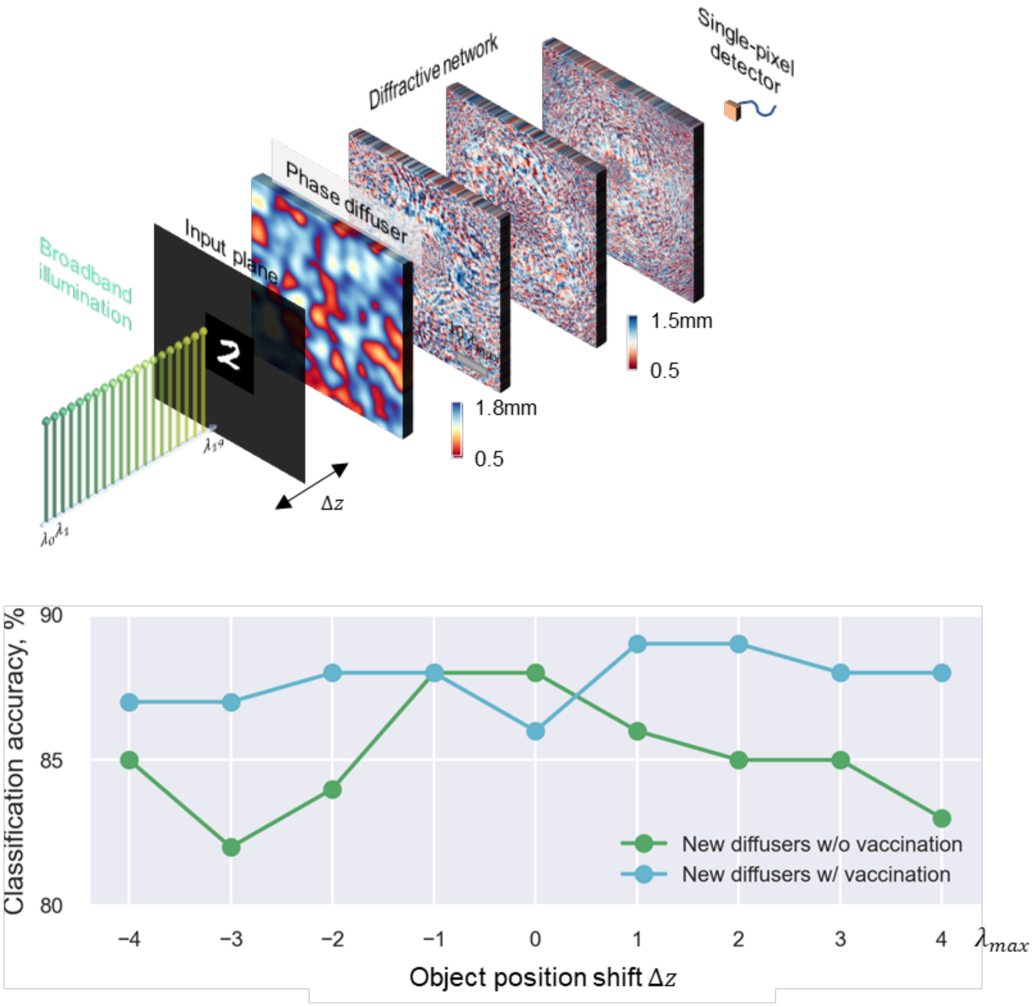

Figure S9. Classification accuracy of single-pixel diffractive neural networks tested with different object-diffuser distances. (a) Schematic of the single-pixel diffractive neural network with different object-diffuser distances. (b) Classification accuracy of the single-pixel diffractive network models trained with/without vaccination using new diffusers. The diffractive network vaccination follows the method described in the Methods section (main text), where Δz is randomly sampled from a uniform distribution $\mathbf{U}(-4\lambda_{max}, 4\lambda_{max})$.

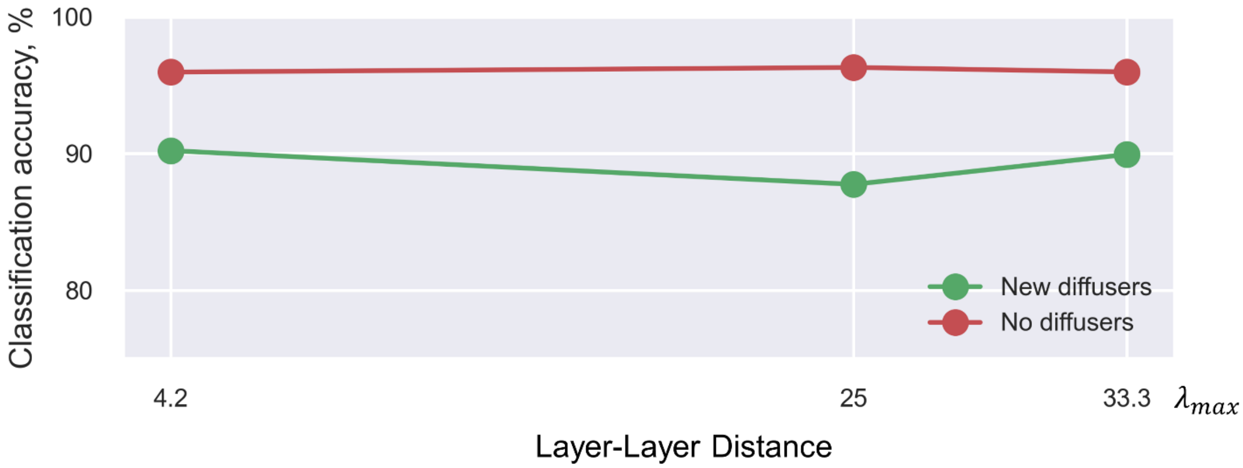

Figure S10. Classification accuracy of single-pixel diffractive neural networks trained with different layer-to-layer distances from $4.2\lambda_{max}$ to $33.3\lambda_{max}$ through new and no diffusers.

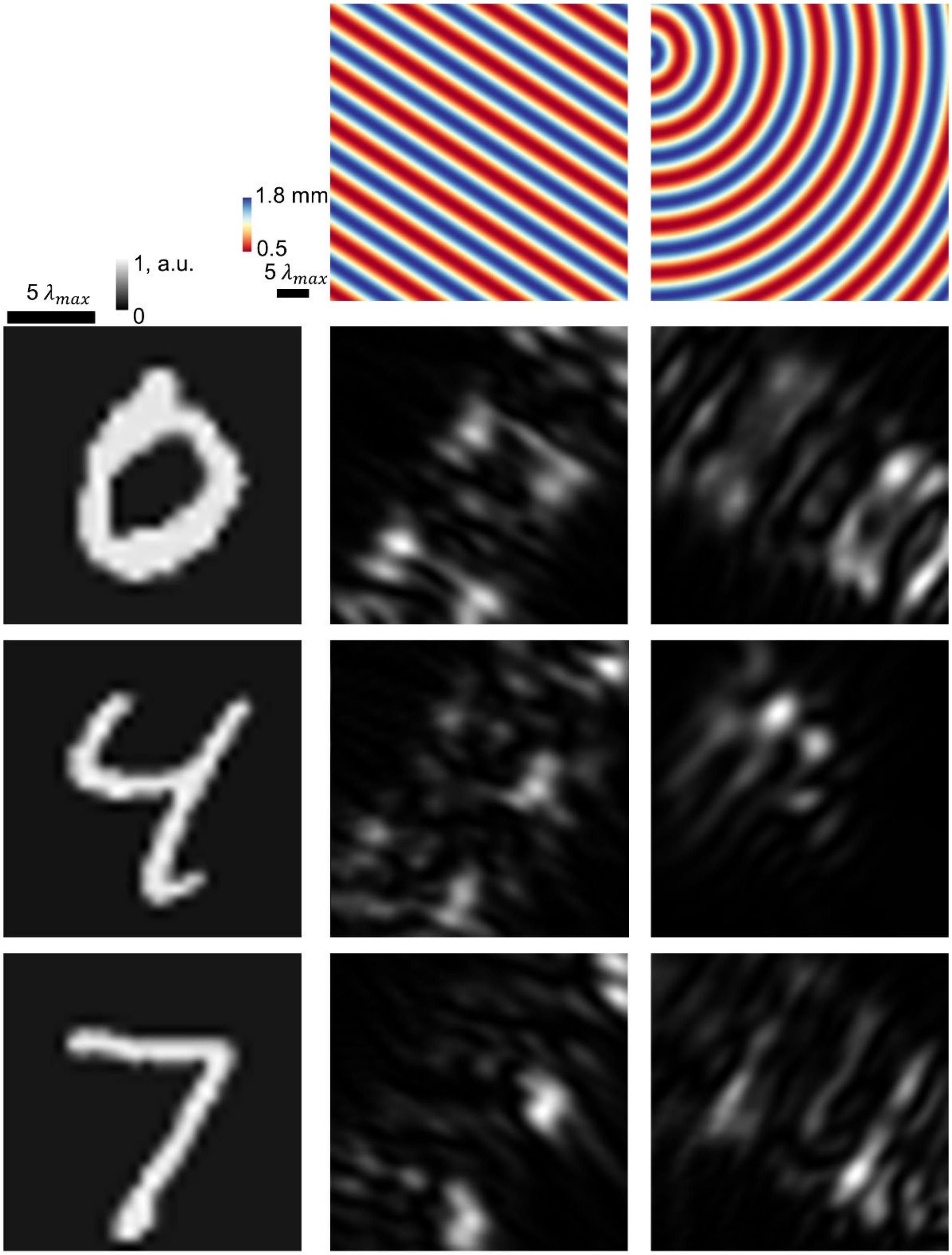

Figure S11. **Different objects seen through linear and circular grating diffusers using a perfect lens-based imaging system.**

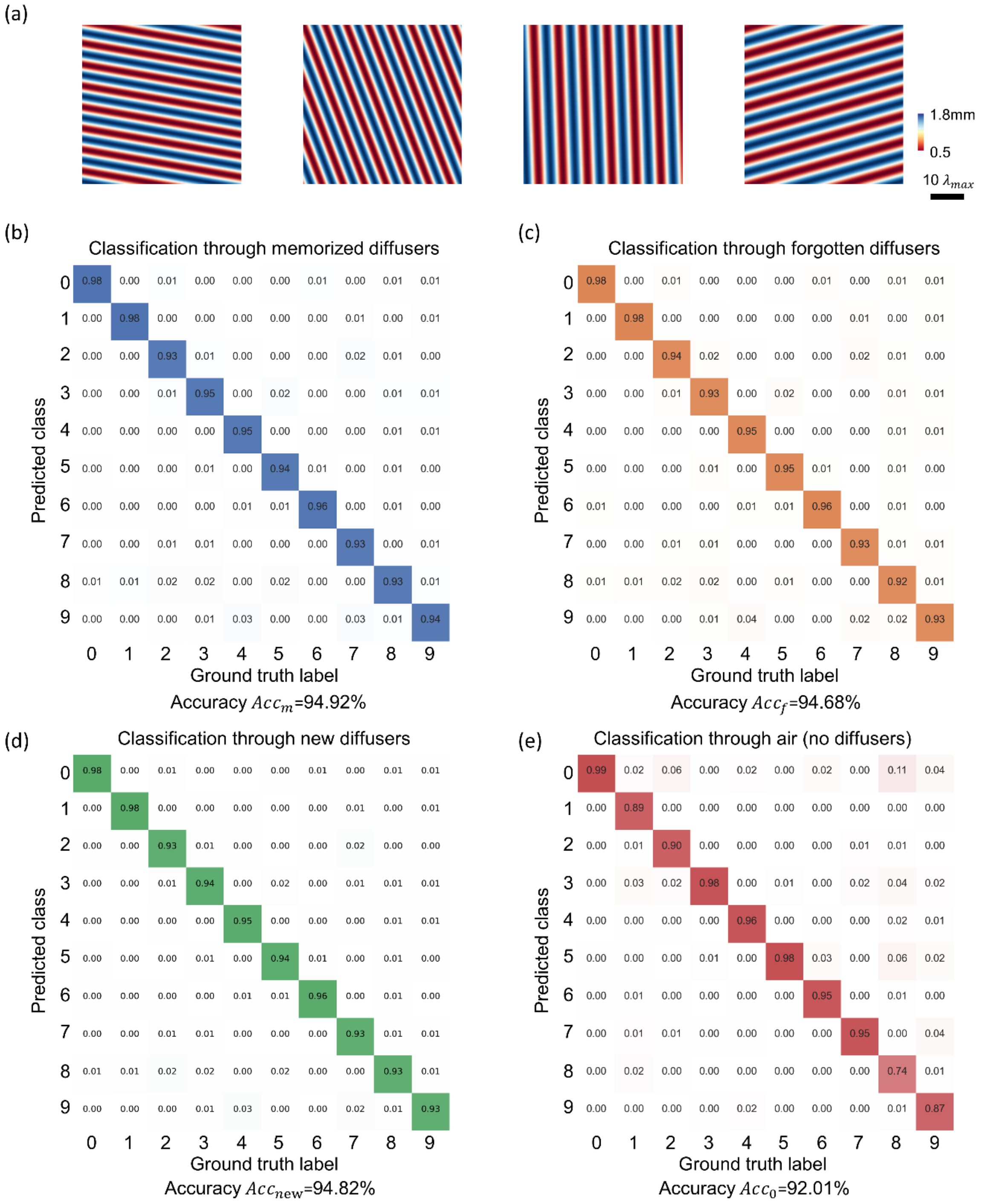

Figure S12. The performance of a single-pixel broadband diffractive network trained to classify unknown objects through unknown linear grating diffusers. (a) Demonstration of linear grating diffusers with different periods and directions. Confusion matrices for the single-pixel diffractive network classifying unknown objects through memorized (b), forgotten (c), new (d), and no diffusers (e), respectively. Notice that $Acc_0 < Acc_m \approx Acc_f \approx Acc_{new} = 94.82\%$ since grating-like diffusers are rather easy for the single-pixel diffractive network to generalize to.

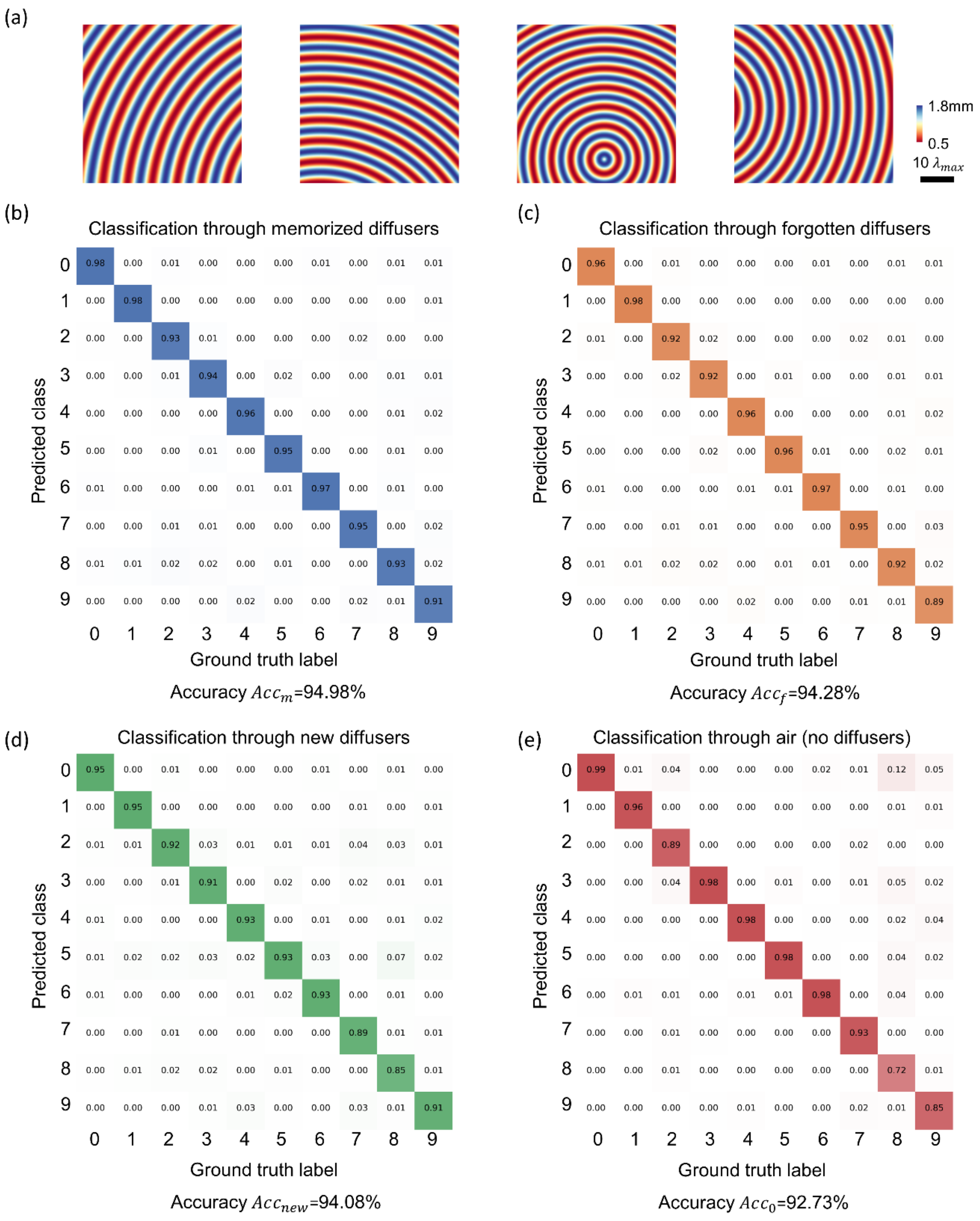

Figure S13. The performance of a single-pixel broadband diffractive network trained to classify unknown objects through unknown circular grating diffusers. (a) Demonstration of circular grating diffusers with different periods and directions. Confusion matrices for the single-pixel diffractive network classifying unknown objects through memorized (b), forgotten (c), new (d), and no diffusers (e), respectively. Notice that $Acc_0 < Acc_m \approx Acc_f \approx Acc_{new} = 94.08\%$ since grating-like diffusers are rather easy for the single-pixel diffractive network to generalize to.
